# Electrical Control of Magnetic Order Transition in 2D Antiferromagnetic Semiconductor FePS_3_

**DOI:** 10.1002/advs.202413892

**Published:** 2025-02-25

**Authors:** Mengjuan Mi, Qing Zhang, Shilei Wang, Xiandong Zhang, Han Xiao, Lixuan Yu, Houning Song, Chao Ma, Shuang Dai, Bingbing lyu, Jiyu Fan, Bing Shen, Fangsen Li, Yanxue Chen, Qing Zhang, Min Liu, Shanpeng Wang, Xiaohui Liu, Yilin Wang

**Affiliations:** ^1^ School of Integrated Circuits Shandong Technology Center of Nanodevices and Integration State Key Laboratory of Crystal Materials Shandong University Jinan 250100 China; ^2^ School of Physics Shandong University Jinan 250100 China; ^3^ State Key Laboratory of Crystal Materials Institute of Crystal Materials Shandong University Jinan 250100 China; ^4^ Shandong Wanbo Technologies Co., LTD Jinan 250100 China; ^5^ Department of Applied Physics Nanjing University of Aeronautics and Astronautics Nanjing 210016 China; ^6^ Center for Neutron Science and Technology and School of Physics Guangdong Provincial Key Laboratory of Magnetoelectric Physics and Devices, and Key Laboratory of Optoelectronic Materials and Technologies Sun Yat‐Sen University Guangzhou 510275 China; ^7^ Vacuum Interconnected Nanotech Workstation Suzhou Institute of Nano‐Tech and Nano‐Bionics Chinese Academy of Sciences Suzhou 215123 China; ^8^ School of Materials Science and Engineering Peking University Beijing 100871 China

**Keywords:** 2D magnetic materials, electrical control of magnetism, magnetic order transition, organic cations intercalation

## Abstract

Manipulating the magnetic order transition of 2D magnetic materials is an important way for the application of spintronic devices, and carrier concentration modulation is a commonly used effective regulation method. Here the magnetic ground state of FePS_3_ is tuned from antiferromagnetic (AFM) to ferrimagnetic (FIM) and back to AFM by electron doping, which is achieved via the intercalation of various organic cations. The doped FePS_3_ with FIM order exhibits a Curie temperature *T*_c_ of ≈110 K, a strong out‐of‐plane magnetic anisotropy, and particularly an unusual hysteresis loop, where with increasing temperature, the area of magnetic hysteresis loop increases below 50 K, then decreases above 50 K and eventually disappears. Theoretical calculations indicate that at a doping concentration of 0.3–0.9 electrons per cell, spin splitting of energy bands occurs, leading to the FIM order; whereas at a doping concentration of ≥ 1.0 electrons per cell, the AFM order recovers. Such AFM‐FIM‐AFM transition is ascribed to the competition between the Stoner exchange‐dominated FM order and super‐exchange‐dominated AFM order. These results demonstrate an effective approach to engineering magnetism in 2D magnetic materials by purely electrical means for future device applications.

## Graphical abstract

The magnetic order transition of FePS_3_ from antiferromagnetic (AFM) to ferrimagnetic (FIM) and back to AFM is achieved via electron doping induced by various organic cations intercalation. The carrier doping‐dependent magnetic transition offers an effective approach to engineering magnetism in 2D magnetic materials by purely electrical means for future device applications.

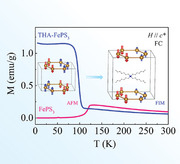

## 1 Introduction

2D magnetism has been studied for many years, beginning with the theoretical prediction of monolayer Ising magnets in 1944.^[^ ^1^ ^]^ However, according to the Mermin–Wagner theorem,^[^ ^2^ ^]^ the long‐range magnetic order has been thought to be difficult to survive in isotropic 2D systems due to thermal fluctuations, but can be stabilized by the magnetic anisotropy revealed by the recent discovery of intrinsic ferromagnetic (FM) order in van der Waals (vdW) crystals, such as Cr_2_Ge_2_Te_6_^[^ ^3^ ^]^ and CrI_3_.^[^ ^4^ ^]^ This significant breakthrough sparked the extensive research of 2D magnets with diverse ground states, which offer an excellent platform to study low‐dimensional magnetism and to explore exotic quantum phases and potential applications in magneto‐electric, magneto‐optic, and spintronic devices, etc.^[^ ^5^, ^6^, ^7^ ^]^

Recently, antiferromagnetic (AFM) materials gain lots of attentions due to their advantages including negligible stray field, robustness against magnetic perturbation, and ultrafast spin dynamics, etc.^[^ ^8^ ^]^ Manipulating the phase transitions of 2D AFM can induce a number of emerging physical phenomena, which is crucial in condensed matter physics and is in great importance in modern information technology.^[^ ^9^ ^]^ For example, the magnetic order transition between AFM and FM enables new types of magneto‐resistive device.^[^ ^10^, ^11^ ^]^ Several methods have been utilized to tune such magnetic order transitions, including magnetic field,^[^ ^12^, ^13^ ^]^ electrical field,^[^ ^14^, ^15^ ^]^ electrostatic doping,^[^ ^16^ ^]^ pressure,^[^ ^9^, ^17^, ^18^ ^]^ etc. Notably, current research mainly focuses on the magnetic order transitions of A‐type AFM materials with intralayer FM and interlayer AFM couplings, such as CrI_3_,^[^ ^4^, ^16^ ^]^ CrCl_3_,^[^ ^19^ ^]^ CrPS_4_^[^ ^12^ ^]^ and CrSBr,^[^ ^13^, ^20^ ^]^ which can be directly detected by optical techniques (e.g., magneto‐optic Kerr effect (MOKE)) and electrical measurements (e.g., spin‐filtering effect). However, it is difficult to directly detect the magnetic order transitions of the intralayer AFM materials with zero net magnetization. Among the intralayer AFM materials, transition metal phosphorous trichalcogenides (MPX_3_, M = Mn, Fe, Ni; X = S, Se) attract lots of interest due to their rich magnetic structures covering three basic models that describe the magnetic coupling of 2D magnetic materials: FePS_3_ of Ising‐type, NiPS_3_ of XY or XXZ‐type, and MnPS_3_ of Heisenberg‐type. In addition, their magnetic orders persist down to the ultrathin limit.^[^ ^21^, ^22^, ^23^, ^24^, ^25^, ^26^, ^27^ ^]^

In this study, we focus on FePS_3_ which exhibits many intriguing physical phenomena, such as terahertz field‐induced metastable magnetization,^[^ ^28^ ^]^ strong magnon‐phonon coupling,^[^ ^29^, ^30^ ^]^ metal‐to‐insulator transition under pressure,^[^ ^31^, ^32^ ^]^ and pressure induced superconductivity in its homologue FePSe_3_.^[^ ^32^ ^]^ These observations motivate further exploration for novel quantum phenomena of FePS_3_. Although it has been theoretically predicted that tensile, compressive strain, and carrier doping could induce an AFM‐FM transition,^[^ ^33^, ^34^ ^]^ carrier doping dependence of magnetic order transition in FePS_3_ and the underlying mechanism are scarce.^[^ ^35^ ^]^

Here, we reported the controllable magnetic order transition from AFM to ferrimagnetic (FIM) in FePS_3_ by electron doping via the organic cations intercalation. Intercalation, known as a process of inserting guest species into the vdW gaps of a host material accompanied with charge doping, has proven to be an effective route for modulating the 2D magnetic materials.^[^ ^36^, ^37^, ^38^, ^39^, ^40^ ^]^ For example, Cr_2_Ge_2_Te_6_ intercalated with tetrabutyl ammonium (TBA^+^) exhibits significantly enhanced *T*_c_, rising from 67 K in pristine Cr_2_Ge_2_Te_6_ to 208 K, which is attributed to the change of magnetic coupling from a weak super‐exchange interaction to a strong double‐exchange interaction through electron doping.^[^ ^36^ ^]^ NiPS_3_ intercalated with TBA^+^ realizes a transition from AFM to FIM with a *T*_c_ = 78 K, which is attributed to the reduction of Ni(ii) → Ni^0^, resulting in the generation of unpaired spins.^[^ ^39^ ^]^ MnPS_3_ intercalated with different organic cations also realizes an AFM‐FIM transition, which is attributed to the generation of different populations of Mn^2+^ vacancies and thus unpaired spins.^[^ ^40^ ^]^ Organic cation intercalation can regulate magnetism well but with different mechanisms, therefore comprehensive and in‐depth research continues to be actively conducted.

Taking advantage of the chemical diversity of organic molecules, various organic cations lead to different doping levels. We found the magnetic ground states of tetraheptyl ammonium (THA^+^) intercalated FePS_3_ (denoted as THA‐FePS_3_) exhibit a FIM order with a Curie temperature *T*_c_ of 110 K, while the magnetic ground states of cetyltrimethyl ammonium (CTA^+^), tetrapropyl ammonium (TPA^+^) and TBA^+^ intercalated FePS_3_ still remain AFM order but with decreased Néel temperature (*T*_N_). Compared with previous reports of 2D magnets intercalation, such as NiPS_3_,^[^ ^39^ ^]^ MnPS_3_,^[^ ^40^ ^]^ and FePS_3_,^[^ ^41^, ^42^, ^43^ ^]^ the THA‐FePS_3_ exhibits a strong out‐of‐plane magnetic anisotropy, largest remanence and coercivity, and particularly an unusual hysteresis loop, where with increasing temperature, the area of magnetic hysteresis loop increases below 50 K, then decreases above 50 K and eventually disappears. Based on the first‐principles calculations, it is found that the AFM‐FIM‐AFM magnetic order transition of FePS_3_ regulated by carrier concentration is caused by the competition between the Stoner exchange‐dominated FM order and super‐exchange‐dominated AFM order.

## 2 Results and Discussion

### 2.1 Intercalation of FePS_3_ Bulk Crystals with THA^+^ Cations

**Figure 1:**
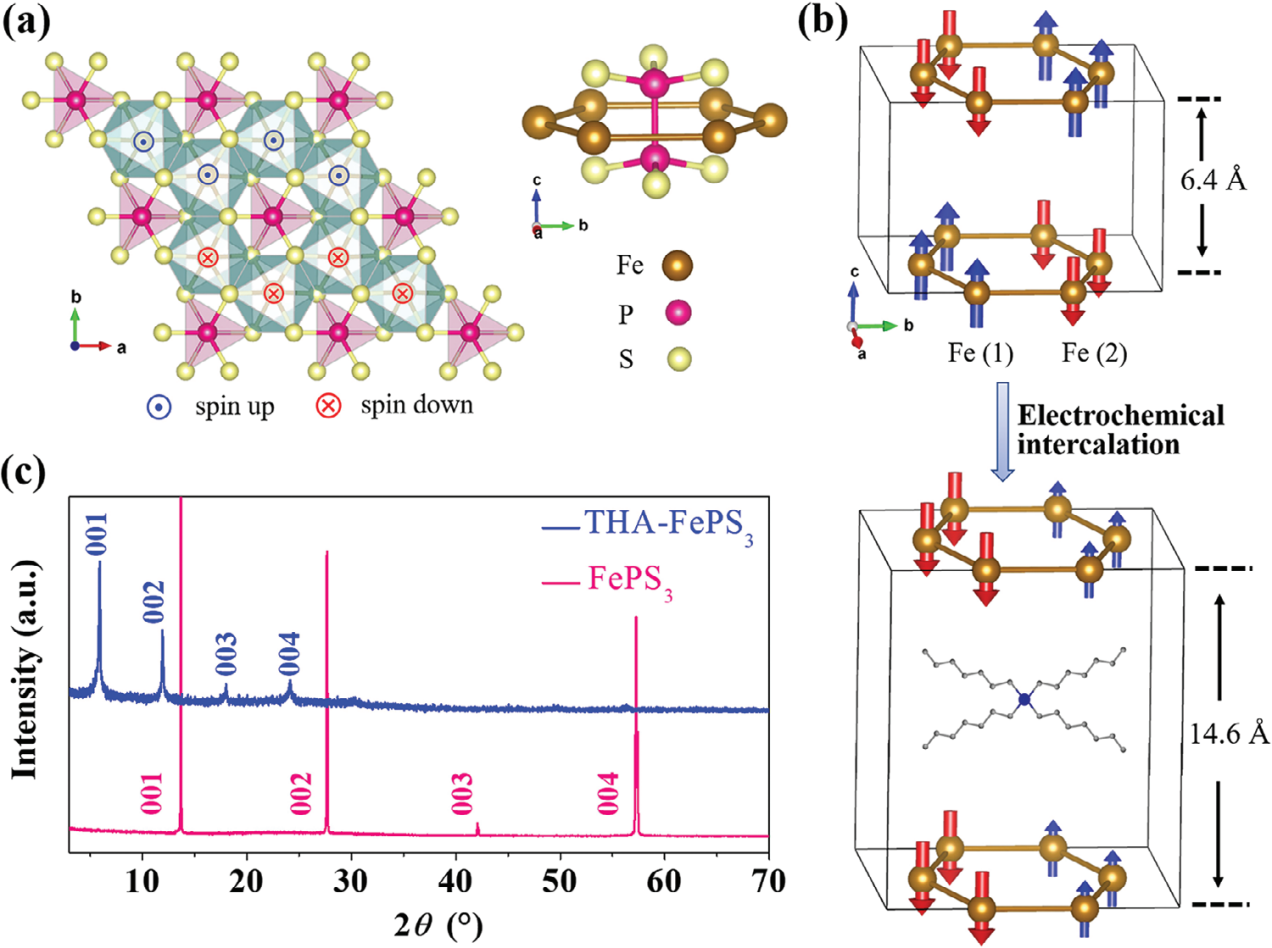
Crystal structure of FePS_3_ and intercalated FePS_3_. a) Crystal structure of FePS_3_. b) Magnetic structures of FePS_3_ (top) and intercalated THA‐FePS_3_ (bottom). The blue and red arrows indicate the direction of magnetic moments. c) XRD patterns of pristine FePS_3_ and intercalated THA‐FePS_3_.

High‐quality FePS_3_ single crystals were used as starting material to prepare organic cations (e.g., THA^+^) intercalated samples. **Figure** **1**a,b show the crystal and magnetic structures of FePS_3_. Bulk FePS_3_ adopts a monoclinic structure with the space group *C*2/*m*. The Fe^2+^ ions form a honeycomb lattice in the *ab* plane, and the two P atoms are oriented along the direction perpendicular to the *ab* plane and covalently attached to six S atoms, forming a [P_2_S_6_]4^−^ unit located in the center of each honeycomb ring.^[^ ^44^ ^]^ The magnetic moments on Fe^2+^ ions are oriented perpendicular to the *ab* plane and coupled ferromagnetically with two of the three nearest neighbors to form zigzag ferromagnetic chains along the *a*‐axis. The two ferromagnetic chains (labeled as Fe(1) and Fe(2) in Figure 1b) are antiferromagnetically coupled along the *b*‐axis.^[^ ^45^ ^]^ The adjacent layers are antiferromagnetically coupled by weak vdW interactions with an interlayer distance of 6.4 Å, making the FePS_3_ easy to be intercalated. After intercalation, the XRD pattern (Figure 1c, blue line) of THA‐FePS_3_ shows only a series of (00l) peaks with lower angles, demonstrating an expanded interlayer distance of 14.6 Å (Figure 1b). For the concentration of THA^+^ cations in intercalated THA‐FePS_3_, a stoichiometric molar ratio of (THA)_0.21_FePS_3_ was estimated based on the gravimetric analysis (TGA) (Figure S1, Supporting Information).

### 2.2 Ferro/Ferrimagnetism in Intercalated THA‐FePS_3_

**Figure 2:**
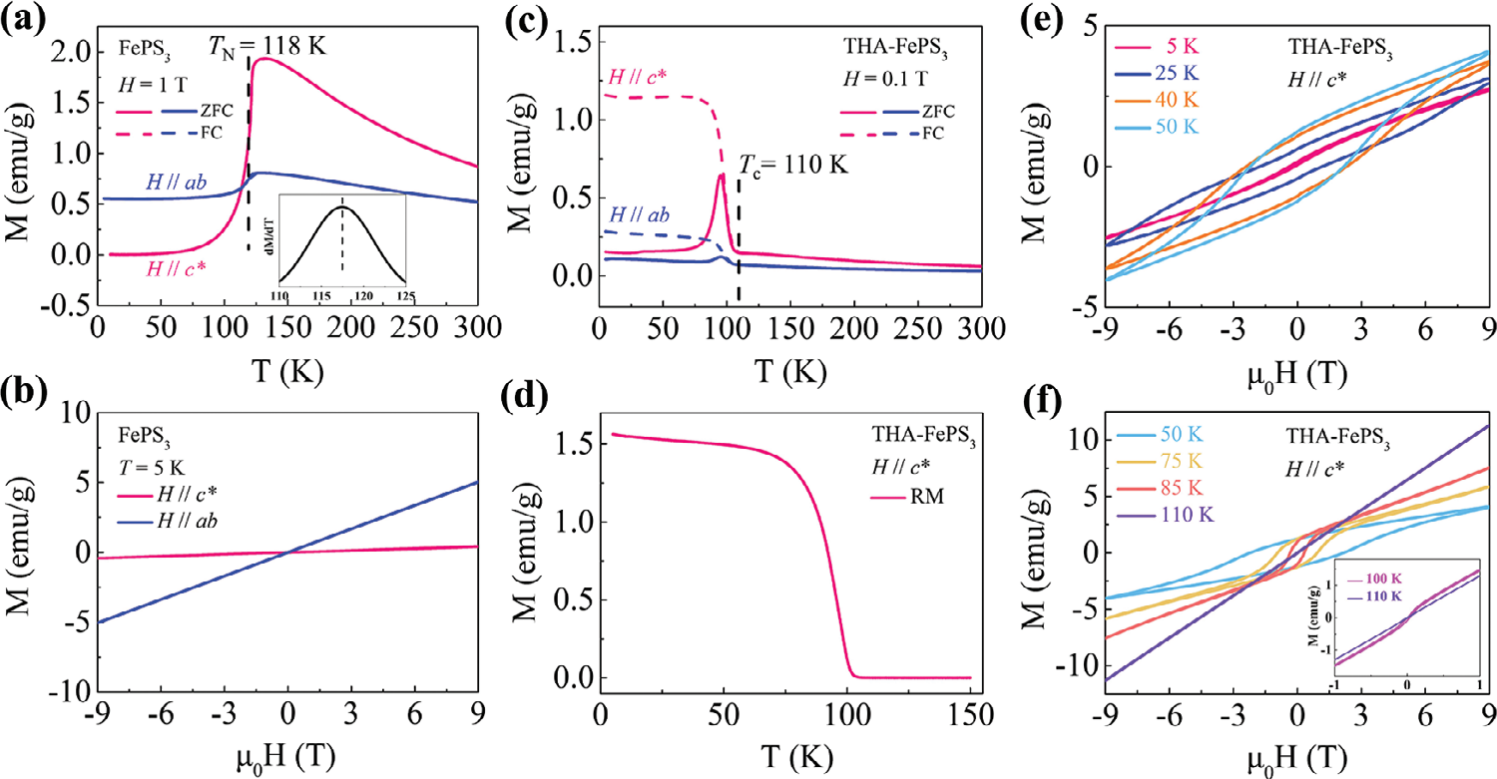
Magnetic properties of pristine FePS_3_ and intercalated THA‐FePS_3_. a,b) Magnetization versus temperature (*M*‐*T*, a) and magnetic field *μ*_0_ *H* (*M*‐*H*, b) of pristine FePS_3_ under magnetic fields *H* // *ab* (blue) and *H* // c* (pink). The solid and dashed lines in (a) represent zero‐field‐cooled (ZFC) and field‐cooled (FC) data, respectively. The inset in (a) shows the first‐order derivative of magnetization with temperature (d*M*/d*T* versus *T*). c) Magnetization versus temperature (*M*‐*T*) of intercalated THA‐FePS_3_ under magnetic fields *H* // *ab* (blue) and *H* // c* (pink). d) The remnant magnetization (RM) was measured during warming under zero field after being cooled under *μ*_0_ *H* = 1 T with H // c*. e,f) Magnetization versus magnetic field *μ*_0_ *H* (*M*‐*H*) at different temperatures under magnetic fields *H* // c*. The inset of (f) shows the zoom‐in image of the *M*‐*H* curves at 100 and 110 K.

To compare the magnetic properties of the pristine FePS_3_ and the intercalated THA‐FePS_3_, temperature‐dependent zero field cooling (ZFC) and field cooling (FC) magnetization (*M*‐*T*) measurements were first performed, where the applied magnetic fields are parallel (*H* // *ab*) or perpendicular (*H* // c*) to the *ab* plane, respectively. As shown in **Figure** **2**a, the pristine FePS_3_ shows AFM characteristics, with a clear magnetic phase transition from the paramagnetic (PM) to AFM at ≈118 K, which is defined from the first derivative d*M*/d*T* and is consistent with previous studies.^[^ ^23^, ^24^, ^44^ ^]^ The broad maximum above *T*_N_ in the *M*‐*T* curve for both *H* // *ab* and *H* // c* is related to short‐range, spin‐spin correlation, which is typical for low‐dimensional magnetic systems.^[^ ^44^, ^46^, ^47^ ^]^ Moreover, a strong magnetic anisotropy was observed, the magnetization changes more significantly in *H* // c* than in *H* // *ab*, indicating the magnetic moments are mainly oriented in an out‐of‐plane direction. Figure 2b shows the magnetization versus magnetic field *μ*_0_ *H* (*M*‐*H*) data for *H* // *ab* and *H* // c* at *T* = 5 K, the linear curves display the typical features of AFM.

After intercalation, the magnetic behaviors of intercalated THA‐FePS_3_ significantly differ from the pristine FePS_3_ counterpart. As shown in Figure 2c, the *M*‐*T* curves for THA‐FePS_3_ in both *H* // *ab* and *H* // c* have a similar trend, and reveal a transition from PM to FM/FIM order (indicating the presence of net magnetic moments ΔM, discuss later) accompanied with *T*_c_ of ≈110 K.

At low temperatures, the magnetization under ZFC is smaller than the magnetization under FC, and the magnetization in H // c* is much greater than in *H // ab*, indicating that the magnetic easy axis of THA‐FePS_3_ remains perpendicular to the *ab* plane. For FC curves, the magnetization remains almost constant below 80 K and then decreases rapidly with increasing temperature, manifesting FM/FIM characteristics. While with increasing temperature, the magnetization in the ZFC curve first increases slowly below 80 K, then increases rapidly until coinciding with the FC curve at ≈100 K, and later decreases rapidly similar to the FC curve. Under different field cooling conditions, ZFC and FC curves exhibit similar trends (Figure S2, Supporting Information).

After intercalation, the interlayer distance expands to 14.6 Å, and thus the interlayer interaction is negligible. During the FC process, the net magnetic moments ΔM of each layer (discuss later) can be aligned by the field, resulting in a large and saturated magnetization at low temperatures. However, during the ZFC process, the ΔM of each layer is randomly oriented (pointing upward or downward with respect to the *ab* plane), leading to a small magnetization at low temperatures. Upon increasing temperature, the magnetic moments opposite to the direction of the magnetic field tend to be aligned by the external magnetic field with the assistance of thermal fluctuations until reaching a maximum (the position at which the FC and ZFC curves almost coincide), and then rapidly decreases due to the thermal fluctuations induced random orientation of magnetic moments, which accounts for the overlapped portion between ZFC and FC curves.

Figure 2d shows the remanent magnetization (RM) of THA‐FePS_3_ measured during warming under zero field after being cooled under *μ*_0_ *H* = 1 T with H // c*. The RM first decreases gradually with increasing temperature, then decreases rapidly at 80 K and becomes zero at *T*≈110 K, indicating the existence of spontaneous magnetization after THA^+^ intercalation.

Figure 2e,f displays the *M*‐*H* curves of THA‐FePS_3_ with H // c* at different temperatures. Below 50 K, as the temperature increases, the hysteresis loops become more pronounced, the area of the hysteresis loop increases, and the RM also increases. Above 50 K, the hysteresis loops decrease as the temperature increases, exhibiting normal behavior consistent with typical FM/FIM materials, and finally disappear and become linear at *T* = 110 K (the inset in Figure 2f), indicating the FM/FIM‐PM transition with *T*_c_ of 110 K. Such unusual features of the hysteresis loops below 50 K are attributed to the extremely large coercive field (*H*_c_ = 38 T) of FePS_3_.^[^ ^30^ ^]^ After intercalation, the THA‐FePS_3_ still retains a significant coercive field, which prevents the magnetic moments from being fully aligned by the applied magnetic field up to 9 T in our measurements at low temperatures. As the temperature increases, thermal kinetic energy favors aligning the magnetic moments parallel to the direction of the magnetic field. The trade‐off between thermal fluctuations and Zeeman energy results in the most pronounced hysteresis loop and the maximum RM at 50 K. For *H* // *ab*, as shown in Figure S3 (Supporting Information), the *M*‐*H* curves of THA‐FePS_3_ have the same trend but with small hysteresis loops, further suggesting that its magnetization easy axis is perpendicular to the *ab* plane.

### 2.3 Properties of FePS_3_ Intercalated with Different Organic Cations

**Figure 3:**
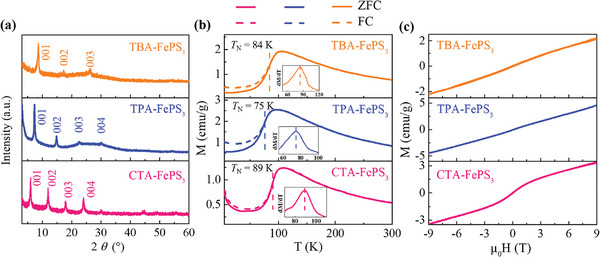
XRD patterns (a), magnetization versus temperature (*M*‐*T*) with *H* = 1 T (b), and magnetization versus magnetic field (*M*‐*H*) at 5 K (c) with *H* // c* of intercalated TBA‐FePS_3_, TPA‐FePS_3_, and CTA‐FePS_3_. The inset in each panel of (b) shows the d*M*/d*T* versus *T*.

In addition to the intercalation of THA^+^ cations, the magnetic properties of FePS_3_ were also regulated by intercalating other organic homologue with different sizes, such as TBA^+^, TPA^+,^ and CTA^+^ cations. **Figure** **3**a shows the XRD patterns of different organic cations intercalated FePS_3_, similar to THA‐FePS_3_, the interlayer distances are expanded to 10.2, 11.7, and 14.8 Å for TBA‐FePS_3_, TPA‐FePS_3,_ and CTA‐FePS_3_, respectively. The concentration of TBA^+^, TPA^+,^ and CTA^+^ cations in intercalated TBA‐FePS_3_, TPA‐FePS_3,_ and CTA‐FePS_3_ determined by the TGA (Figure S4, Supporting Information) is estimated to be 0.23, 0.24, and 0.31, respectively. To investigate the systematic changes of the magnetic properties of different organic cations intercalated FePS_3_, the *M*‐*T* and *M*‐*H* curves with *H* // c* were measured, and the results are shown in Figure 3b,c. The *M*‐*T* curves of TBA‐FePS_3_, TPA‐FePS_3_, and CTA‐FePS_3_ are similar to that of the pure FePS_3_ counterpart (Figure 2a), an AFM order is still maintained but with lowered *T*_N_, corresponding to 84, 75, and 89 K, respectively. At temperatures below 30 K, the magnetization of CTA‐FePS_3_ starts to increase with decreasing temperature, which may originate from the uncompensated magnetic moments in certain regions caused by non‐uniform intercalation.^[^ ^48^ ^]^ Such phenomenon was also manifested in *M*‐*H* curves, CTA‐FePS_3_ shows an S‐shaped hysteresis at low temperature and the hysteresis gradually disappears with the increase of temperature up to 50 K (Figure S5, Supporting Information).

### 2.4 Characterization of Intercalated FePS_3_

**Figure 4:**
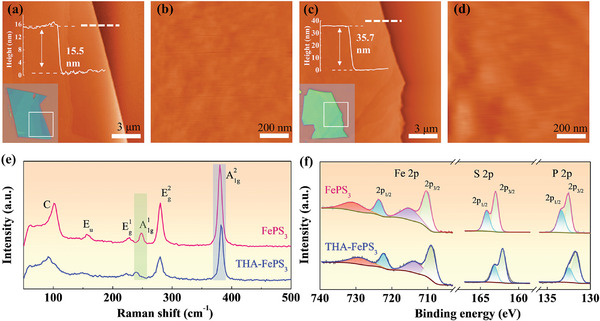
a–d) AFM topography images and enlarged AFM images of exfoliated pristine FePS_3_ (a, b) and intercalated THA‐FePS_3_ (c, d), respectively. Inserts in (a), (c) are AFM height profiles (top) corresponding to white dashed lines and optical images (bottom) of pristine FePS_3_ and intercalated THA‐FePS_3_, respectively. e,f) Raman (e) and XPS (f) spectra of pristine FePS_3_ and intercalated THA‐FePS_3_, respectively.

To explore the origin why such AFM‐FM/FIM transition occurs in THA‐FePS_3_ while not in other organic cations intercalated FePS_3_, the structural and chemical characterizations were performed. **Figure** **4** shows the AFM topography images of exfoliated pristine FePS_3_ and intercalated THA‐FePS_3_, AFM images of other organic cations intercalated FePS_3_, e.g. CTA‐FePS_3_, TBA‐FePS_3_ are shown in Figure S6 (Supporting Information). Both pristine and intercalated FePS_3_ have clean and smooth surfaces without obvious defects, and the root‐mean‐square (RMS) roughness merely increases from 0.14 nm for pristine FePS_3_ to 0.21, 0.28, and 0.24 nm for THA‐FePS_3_, CTA‐FePS_3_, and TBA‐FePS_3_, respectively. In addition, a step of 1.63 nm is observed in THA‐FePS_3_, which matches well with the XRD results (Figure S7, Supporting Information).

Raman spectroscopy measurements were also performed to verify whether defects are formed after intercalation. Figure 4e shows the Raman spectra of pristine FePS_3_ and intercalated THA‐FePS_3_. For pristine FePS_3_, several modes corresponding to the vibrations of Fe^2+^ cations (C mode) and molecular [P_2_S_6_]^4−^ units (infrared active E_u_ mode, $A_{1g}^{1}$ and $\;A_{1g}^{2}$ modes for out‐of‐plane vibration, and $E_{g}^{1}$ and $E_{g}^{2}$ modes for in‐plane vibration) were observed, which is consistent with previous results.^[^ ^49^, ^50^ ^]^ On the country, for intercalated THA‐FePS_3_, all the Raman characteristic peaks of pristine FePS_3_ are maintained but with weakened intensity. The weakened and broadened E_u_ mode indicates the reduced interactions between [P_2_S_6_]^4−^ units along the *c*‐axis.^[^ ^49^, ^51^, ^52^ ^]^ Moreover, the red‐shift of $A_{1g}^{1}$ mode and blue‐shift of $A_{1g}^{2}$ modes are consistent with the change in FePS_3_ as the number of layers decreases (Figure S8, Supporting Information), further confirming the reduced interlayer interactions. The Raman spectra of TBA‐FePS_3_ and CTA‐FePS_3_ are shown in Figure S9 (Supporting Information), showing a similar feature as THA‐FePS_3_. The Raman results indicate that the intercalated FePS_3_ maintains a stable structure, and the intercalation does not destroy the intralayer structure of FePS_3_. High‐resolution transmission electron microscopy (HRTEM) and selected area electron diffraction (SAED) further confirm that there are no noticeable structural distortions, defects or impurity phases in intercalated FePS_3_ (Figure S10, Supporting Information).

X‐ray photoelectron spectroscopy (XPS) was carried out to analyze the elemental composition and chemical state of the intercalated FePS_3_, and the results are shown in Figure 4f. For pristine FePS_3_, the Fe 2*p* spectrum includes peaks located at 709.8 and 723.1 eV corresponding to 2*p*_3/2_ and 2*p*_1/2_ core levels, respectively. The S 2*p* spectrum includes peaks located at 162.8 and 164.0 eV corresponding to 2*p*_3/2_ and 2*p*_1/2_ core levels, respectively. The P 2*p* spectrum includes peaks located at 132.5 and 133.3 eV corresponding to 2*p*_3/2_ and 2*p*_1/2_ core levels, respectively. For THA‐FePS_3_, similar features as pristine FePS_3_ were observed except for that all the 2*p*_3/2_ and 2*p*_1/2_ peaks of Fe, S, and P shift towards lower binding energies, while the magnitude of spin‐orbit splitting (*E*_2_ *_p_*_1/2 –_ *E*_2_ *_p_*_3/2_) remains consistent (Table S1, Supporting Information). The XPS results indicate that the intercalated THA‐FePS_3_ is electron‐doped, and the intercalation does not induce impurity phases.

### 2.5 Mechanism for Magnetic Order Transition in Intercalated FePS_3_

All the intercalated FePS_3_ have enlarged interlayer distance depending on the size and arrangement of organic cations, while having different magnetic orders. For example, the CTA‐FePS_3_ has an interlayer distance close to the THA‐FePS_3_, however, the CTA‐FePS_3_ exhibits AFM characteristics, whereas the THA‐FePS_3_ exhibits FM/FIM characteristics. The intercalated organic cations are non‐magnetic and non‐conductive, and whether their molecular structure is ordered or disordered does not appear to directly influence the magnetic states. The amount of intercalated organic cations is proportional to the electron doping level, which serves as a key factor in determining the magnetic states. Therefore, first‐principles calculations with Quantum‐ESPRESSO^[^ ^53^ ^]^ were performed to explore the effects of electron doping on the electronic and magnetic properties of FePS_3_.

**Figure 5:**
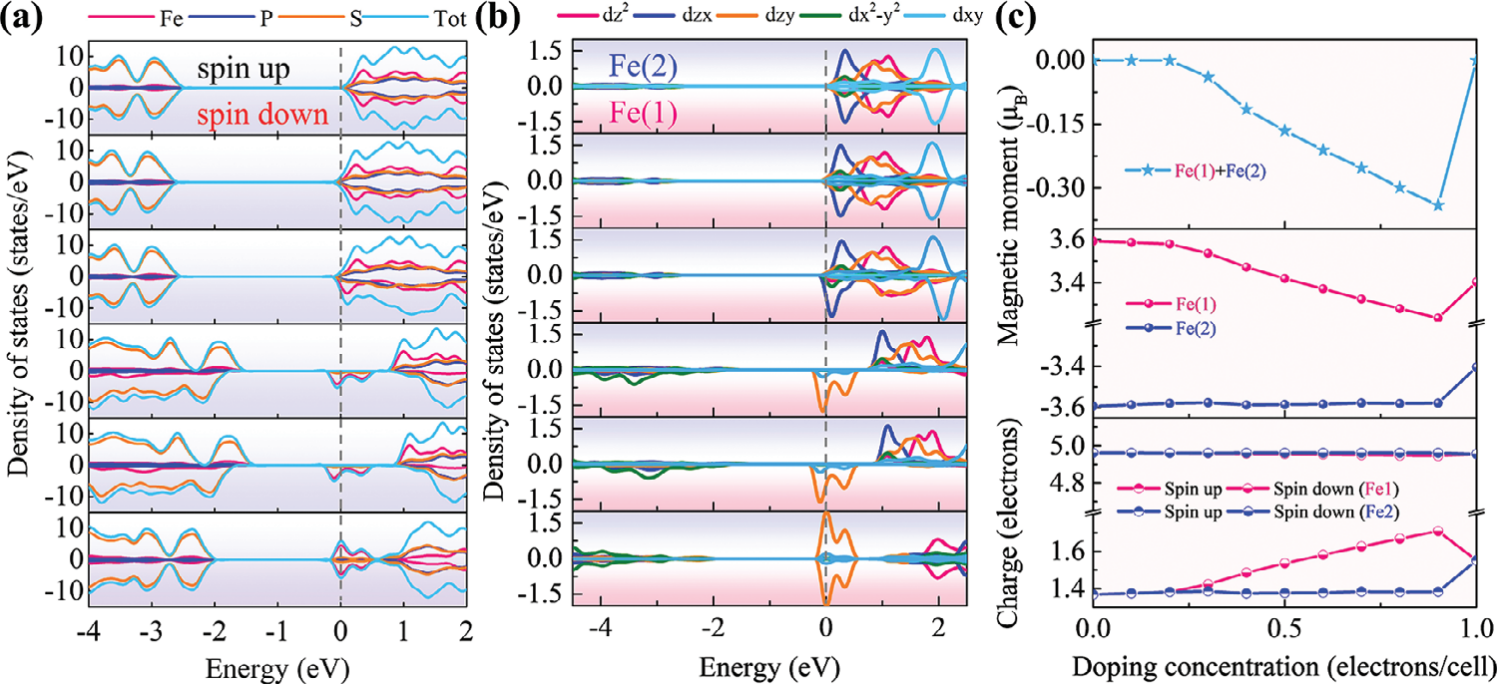
Electronic properties of FePS_3_ at various doping concentrations. a) The PDOS of FePS_3_ at 0, 0.2, 0.3, 0.8, 0.9, and 1.0 electrons per cell from top to bottom. b) The *d* orbital resolved PDOS of Fe atoms (including both Fe(1) and Fe(2)) at 0, 0.2, 0.3, 0.8, 0.9, and 1.0 electrons per cell from top to bottom. c) Net magnetic moments (top), magnetic moments of Fe(1) and Fe(2) (middle), and charges of Fe(1) and Fe(2) with spin‐up and spin‐down configurations (bottom) as a function of doping concentrations. The electron density corresponding to a doping concentration of 0.1 electrons per cell is 1.6 × 10^13^ cm^−2^.

**Figure** **5**a shows the projected density of states (PDOS) of FePS_3_ with different doping levels, and the corresponding band structures are shown in Figure S11 (Supporting Information). For pristine FePS_3_, the energy bands are spin degenerate. With a small amount of doping concentration, e.g., ≤0.2 electrons per cell, the bands are still spin degenerate. After the doping concentration increases to 0.3 electrons per cell, spin splitting of energy bands occurs, and the spin splitting gets larger with increasing doping concentration. Until the doping concentration further increases to 1.0 electrons per cell, the spin splitting disappears, while the splitting of orbitals still exists.

Because the doped electrons occupy the bottom of the conduction band which is primarily composed of Fe‐*d* orbitals, the PDOS for Fe atoms on each ferromagnetic chain with opposite orientations of magnetic moments (Fe(1) site and Fe(2) site shown in Figure 1b) at various doping levels are calculated, and the results are shown in Figure 5b. The orbital‐resolved band structures of FePS_3_ at different doping concentrations are shown in Figure S12 (Supporting Information). The spin splitting of energy bands causes the doped electrons to preferentially occupy Fe(1) zigzag ferromagnetic chain at a doping concentration range of 0.3 – 0.9 electrons per cell. In addition, the conduction band minimum (CBM) is primarily determined by various *d* orbitals of Fe atoms for pristine FePS_3_, and as the doping concentration increases, the CBM is primarily determined by the *d*_zy_ and *d*_xy_ orbitals, which primarily overlap along the inter‐chain direction, making electrons inter‐chain itinerant. When the concentration of inter‐chain itinerant electrons is sufficiently large, the Stoner effect may be triggered to induce ferromagnetism for the itinerant electrons.

Stoner model considers the competition between kinetic energy and exchange energy, and is suitable for describing the ferromagnetic behavior of self‐doped insulators.^[^ ^54^ ^]^ The calculated total number of *d* electrons of Fe^2+^ ion from PDOS is 6.22 and the magnetic moment of the Fe^2+^ is 3.6 μ_*B*_, which differ from the formal values (6 and 4 μ_*B*_), indicating that FePS_3_ can be regarded as a self‐doped negative charge transfer insulator (Figure S13, Supporting Information). According to the Stoner criterion, when *D*(*E*_F_) × *I* > 1, spin splitting occurs in the band structure, where *D*(*E*_F_) and *I* represent the total DOS at the Fermi level (*E*_F_) and the Stoner parameter, respectively, and *I* is equal to the exchange splitting (Δ) of the spin‐up and spin‐down bands divided by the corresponding magnetization density (m, m = n^↑^−n^↓^, n^↑^(n^↓^) is the total number of electrons in spin up (down) bands).^[^ ^54^, ^55^ ^]^ With a small amount of doping concentration, the small DOS at *E*_F_ doesn't satisfy the Stoner criterion. However, as the doping concentration increases, e.g., 0.3 electrons per cell, *D*(*E*_F_) becomes 5.4 states per eV (Figure S14, Supporting Information), and *I* is 0.67 eV, the Stoner criterion is satisfied, leading to the inter‐chain spin splitting. Therefore, as shown in Figure 5c, the doped electrons merely occupy one single Fe chain, which results in the unequal magnetic moments on Fe(1) and Fe(2) sites, leading to the net magnetic moments and making the suitably doped FePS_3_ exhibit FIM characteristics.

However, as the doping concentration further increases to 1.0 electrons per cell, spin splitting disappears, the net magnetic moments become zero and the system prefers the Néel‐type antiferromagnetic (nAFM) state which has the lowest energy (Table S2, Supporting Information). As the electron concentration further increases to 1.1 electrons per /cell, the energy of the stripe‐type antiferromagnetic (sAFM) state becomes the lowest. The competition between Stoner exchange‐dominated FM order and local magnetic moment‐dominated AFM order determines the ground magnetic order of FePS_3_, leading to the AFM‐FIM‐AFM transition as the doping concentration increases. A quantitative understanding of the magnetic ordering transition at a high doping level needs further exploration in future work.

Because FePS_3_ is a semiconductor, its electrical conductivity may reflect the doping levels.^[^ ^56^ ^]^ The electrical conductivities of pristine FePS_3_, THA‐FePS_3_, TBA‐FePS_3_ and CTA‐FePS_3_ are 5.81 × 10^−5^ S cm^−1^, 5.05 × 10^−4^ S cm^−1^, 6.99 × 10^−3^ S cm^−1^ and 3.25 × 10^−3^ S cm^−1^, respectively (Figure S15, Supporting Information). The conductivities of intercalated FePS_3_ are much higher than pristine FePS_3_, and the conductivities of TBA‐FePS_3_ and CTA‐FePS_3_ are much higher than that of THA‐FePS_3_, indicating that the TBA^+^ and CTA^+^ cations intercalation can induce a greater doping concentration than THA^+^ cations intercalation. The concentrations of THA^+^, TBA^+^, TPA^+,^ and CTA^+^ cations in the intercalated THA‐FePS_3_, TBA‐FePS_3_, TPA‐FePS_3,_ and CTA‐FePS_3_ are 0.21, 0.23, 0.24, and 0.31, respectively. Assuming each intercalated cation contributes one doping electron, the THA‐FePS_3_ falls within the appropriate doping concentration that induces FIM order, while the other samples exceed this range.

The environmental stability of THA‐FePS_3_, including its resistance to humidity and temperature, was also investigated. As shown in Figures S17 and S18 (Supporting Information), the THA‐FePS_3_ demonstrates resistance to humidity and remains thermally stable up to 100 °C.

## 3 Conclusion

In summary, we successfully realized the magnetic order transition of FePS_3_ from AFM to FIM and back to AFM as electron doping increases induced by the intercalation of different organic cations into vdW gaps of FePS_3_. The THA^+^ cations intercalated FePS_3_ shows FIM order with *T*_c_ ≈110 K, whereas the CTA^+^, TPA^+,^ and TBA^+^ cations intercalated FePS_3_ remain AFM order with decreased *T*_N_. All intercalated FePS_3_ have expanded interlayer spacing, whereas the THA^+^ cations lead to a doping level much smaller than the other cations. Theoretical calculations indicate that at a doping concentration of 0.3–0.9 electrons per cell, the doped electrons merely occupy one ferromagnetic chain in FePS_3_ due to the spin splitting of energy bands, which causes unequal magnetic moments on Fe(1) and Fe(2) sites and generates net magnetic moments, such that moderately doped FePS_3_ exhibits FIM characteristics; at a doping concentration of ≤0.2 electrons per cell or ≥ 1.0 electrons per cell, spin splitting disappears, such that the lightly or heavily doped FePS_3_ exhibit AFM characteristics. Such carrier doping tuned magnetic order transition is caused by the competition between Stoner exchange‐dominated FM order and super‐exchange‐dominated AFM order, which offers an effective way to manipulate the magnetic and electronic properties in 2D magnetic materials. Moreover, the tunable magnetic properties of FePS_3_ can modulate the exchange bias effect by changing the exchange coupling strength and pinning effect in heterostructures composed of AFM and FM materials.^[^ ^57^, ^58^, ^59^ ^]^ These properties hold potential for applications in spin‐valve devices and thermally assisted magnetic random access memory.

## 4 Experimental Section

### Sample Preparation

FePS_3_ crystals were grown by the vapor transport method. Stoichiometric mixtures of Fe, P, S (mole ratio Fe:P:S = 1:1:3) with iodine as the transport agent were sealed into a quartz tube and then loaded into a two‐zone furnace with a temperature of 750–700 °C. After 2 weeks, the crystals of FePS_3_ were formed on the cold side of the tube.

### Electrochemical Intercalation

A two‐electrode electrochemical setup was used to perform the intercalation experiment. The fresh FePS_3_ crystal serves as a cathode electrode by being directly fixed on the electrode holder, and a piece of Pt serves as an anode electrode. The tetraheptyl ammonium bromide (THA^+^Br^−^, Macklin, 98%) power in acetonitrile (Macklin, 99%) with a concentration of 5 mg ml^−1^ was used as the electrolyte. In this case, intercalation was driven by applying a voltage swept from 0 to ≈5 V and proceeded for 0–1 h at 50 °C. When the voltage was applied, the bromide ions moved towards the anode, where they lost electrons to form Br_2_. While THA^+^ cations were migrated to the cathode electrode and inserted into the vdW gaps of FePS_3_ to balance the electrons attained from the external circuit:

(1) $$Br^{-}\to \frac{1}{2}Br_{2}+e^{-}$$

(2) $$\mathrm{FeP}S_{3}+x\mathrm{TH}A^{+}+xe^{-}\to {\left(\mathrm{THA}\right)}_{x}\mathrm{FeP}S_{3}$$

The intercalation of cetyltrimethyl ammonium (CTA^+^), tetrapropyl ammonium (TPA^+^) and tetrabutyl ammonium (TBA^+^) share the same procedure as that of THA^+^.

### Characterization

The X‐ray diffraction (XRD) patterns were performed on a SmartLabhigh resolution X‐ray diffractometer (Rigaku, Japan) equipped with Cu *K*_a_ radiation (λ = 1.5418 Å). The thermogravimetric analysis (TGA) was taken on TGA5500 (TA, USA). The X‐ray photoelectron spectroscopy (XPS) spectra were conducted using PHI‐5000 VersaprobeII (Ulvac‐Phi, Japan) with Al *K*_α_ X‐ray (hν = 1486.6 eV). The C─C 1s bond (BE = 284.8 eV) was used for energy referencing. Raman characterization was performed with inVia confocal Raman microscope (Renishaw) using an excitation wavelength of 532 nm. The high‐resolution transmission electron microscopy (HRTEM) and selected area electron diffraction (SAED) images of THA‐FePS_3_ were acquired by a FEI Talos F200X microscope at 200 kV. Magnetization measurements were carried out using the vibrating sample magnetometer option (VSM) of a commercial physical property measurement system (Dynacool‐9, Quantum Design).

### First‐Principles Density Functional Theory (DFT) Calculations

DFT calculations were performed using Quantum ESPRESSO.^[^ ^53^ ^]^ The exchange and correlation effects were treated within the generalized gradient approximation (GGA) of Perdew–Burke–Ernzerhof (PBE).^[^ ^60^ ^]^ PSlibrary ultra‐soft pseudopotential was adopted in the calculation.^[^ ^61^ ^]^ In order to correctly describe van der Waals interactions between layers, the semiempirical DFT‐D method was used.^[^ ^62^ ^]^ The Brillouin zone was sampled with 7 × 4 × 5 Monkhorst‐Pack k‐point meshes for the FePS_3_ with a supercell made up of 20 atoms. The electronic wave functions were expanded in a plane‐wave basis set limited by a cut‐off energy of 680 eV. The convergence criteria for the total energy was less than 10^−6^ eV. Atomic positions and lattice constants were relaxed until the force was less than 10 meV Å^−1^. In order to properly describe the on‐site electronic correlation within Fe 3*d* orbitals, the DFT + U method was adopted and U was set to 7 eV.

## Conflict of Interest

The authors declare no conflict of interest.

## Supporting information



Supporting Information

## Data Availability

The data that support the findings of this study are available from the corresponding author upon reasonable request.
