# Supplementary material for: Electrical Control of Magnetic Order Transition in 2D Antiferromagnetic Semiconductor FePS3
Source: Adv Sci (Weinh). 2025 Feb 25;12(15):2413892. doi: 10.1002/advs.202413892 (PMC12005775; doi:10.1002/advs.202413892)
Supplement: Supplementary file 1 — Supporting Information [file ADVS-12-2413892-s001.docx]

Supporting Information

**Electrical Control of Magnetic Order Transition in** **Two-dimensional Antiferromagnetic Semiconductor FePS_3_**

*Mengjuan Mi,^1#^ Qing Zhang,^2#^ Shilei Wang,^3^ Xiandong Zhang,^4^ Han Xiao,^1^ Lixuan Yu,^1^ Houning Song,^2^ Chao Ma,^3^ Shuang Dai,^4^ Bingbing lyu,^1^ Jiyu Fan,^5^ Bing Shen,^6^ Fangsen Li,^7^ Yanxue Chen,^2^ Qing Zhang,^8^ Min Liu,^1*^ Shanpeng Wang,^3*^ Xiaohui Liu,^2*^ Yilin Wang^1*^*

^1^School of Integrated Circuits, Shandong Technology Center of Nanodevices and Integration, State Key Laboratory of Crystal Materials, Shandong University, Jinan 250100, China

^2^School of Physics, Shandong University, Jinan 250100, China

^3^State Key Laboratory of Crystal Materials, Institute of Crystal Materials, Shandong University, Jinan, 250100, China

^4^Shandong Wanbo Technologies Co., LTD, Jinan 250100, China

^5^Department of Applied Physics, Nanjing University of Aeronautics and Astronautics, Nanjing, 210016, China

^6^Center for Neutron Science and Technology and School of Physics, Guangdong Provincial Key Laboratory of Magnetoelectric Physics and Devices, and Key Laboratory of Optoelectronic Materials and Technologies, Sun Yat-Sen University, Guangzhou 510275, China

^7^Vacuum Interconnected Nanotech Workstation, Suzhou Institute of Nano-Tech and Nano-Bionics, Chinese Academy of Sciences, Suzhou 215123, China

^8^School of Materials Science and Engineering, Peking University, Beijing 100871, China

^#^These Authors contributed equally to this work


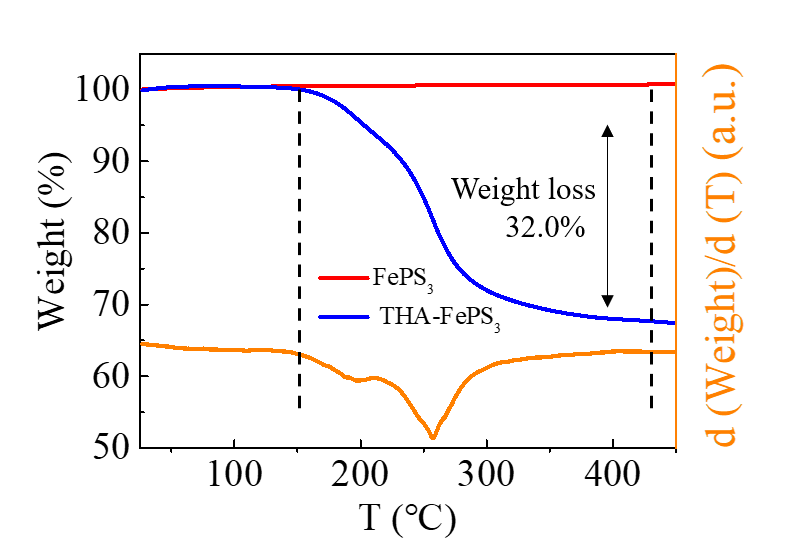


**Figure S1.** The TGA for both pristine FePS_3_ and intercalated THA-FePS_3_. The temperature range was 25 ℃ - 450 ℃ and the whole experiment was conducted under Nitrogen gas at a heating rate of 5 ℃ /min.


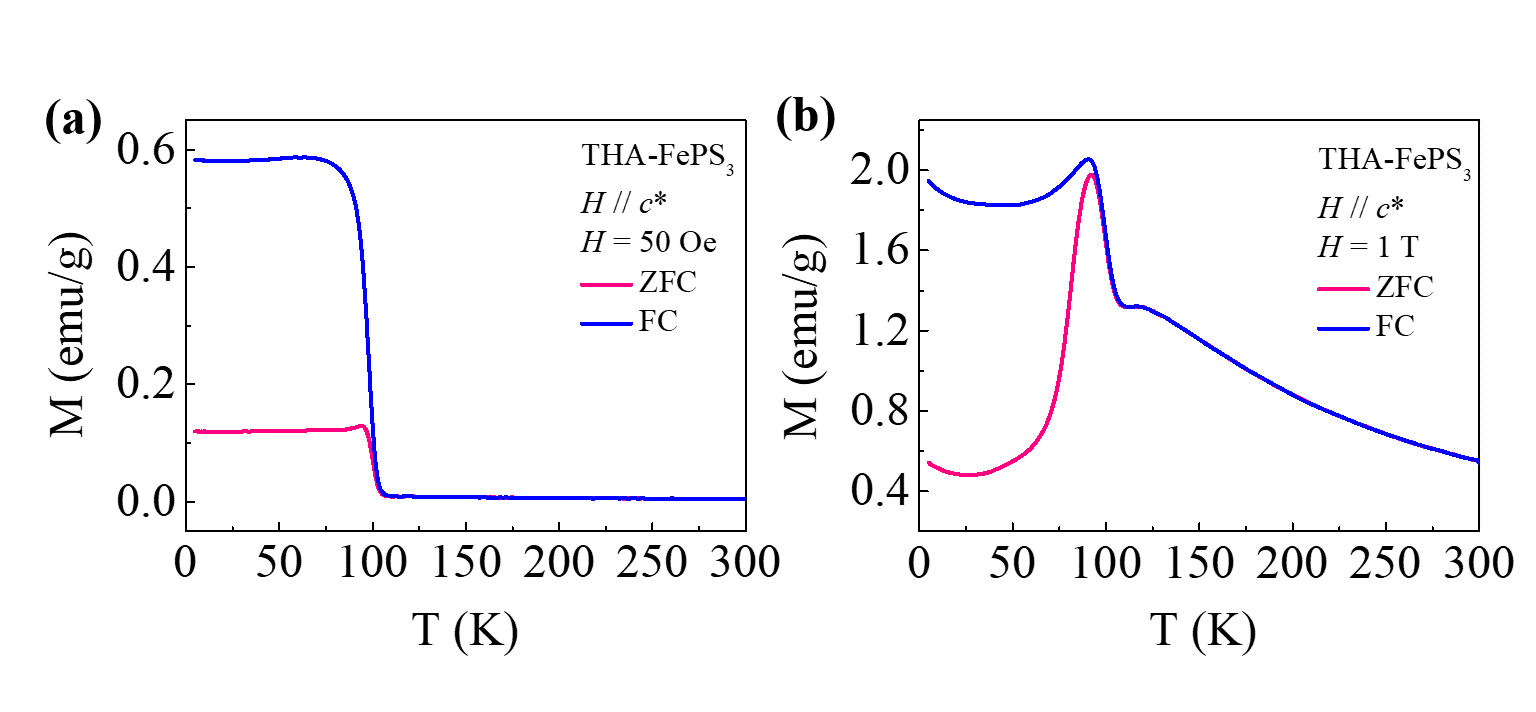


**Figure S2.** Magnetization versus temperature (*M*-*T*) under magnetic fields *H* // *c** of 50 Oe (a) and 1 T (b), respectively.


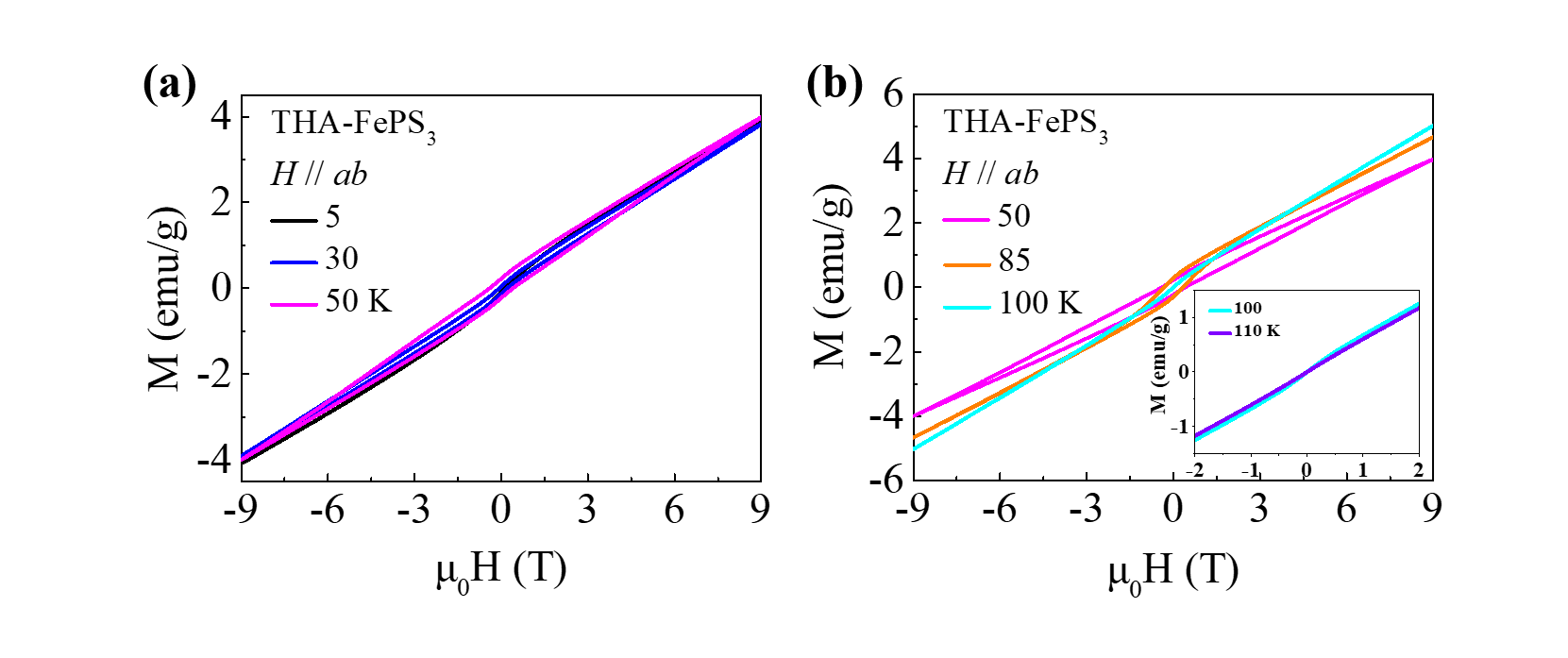


**Figure S3.** Isothermal magnetization of intercalated THA-FePS_3_ under magnetic field *H* // *ab* at different temperatures. The inset of (b) shows the zoom-in image of the *M*-*H* curves of 100 K and 110 K.


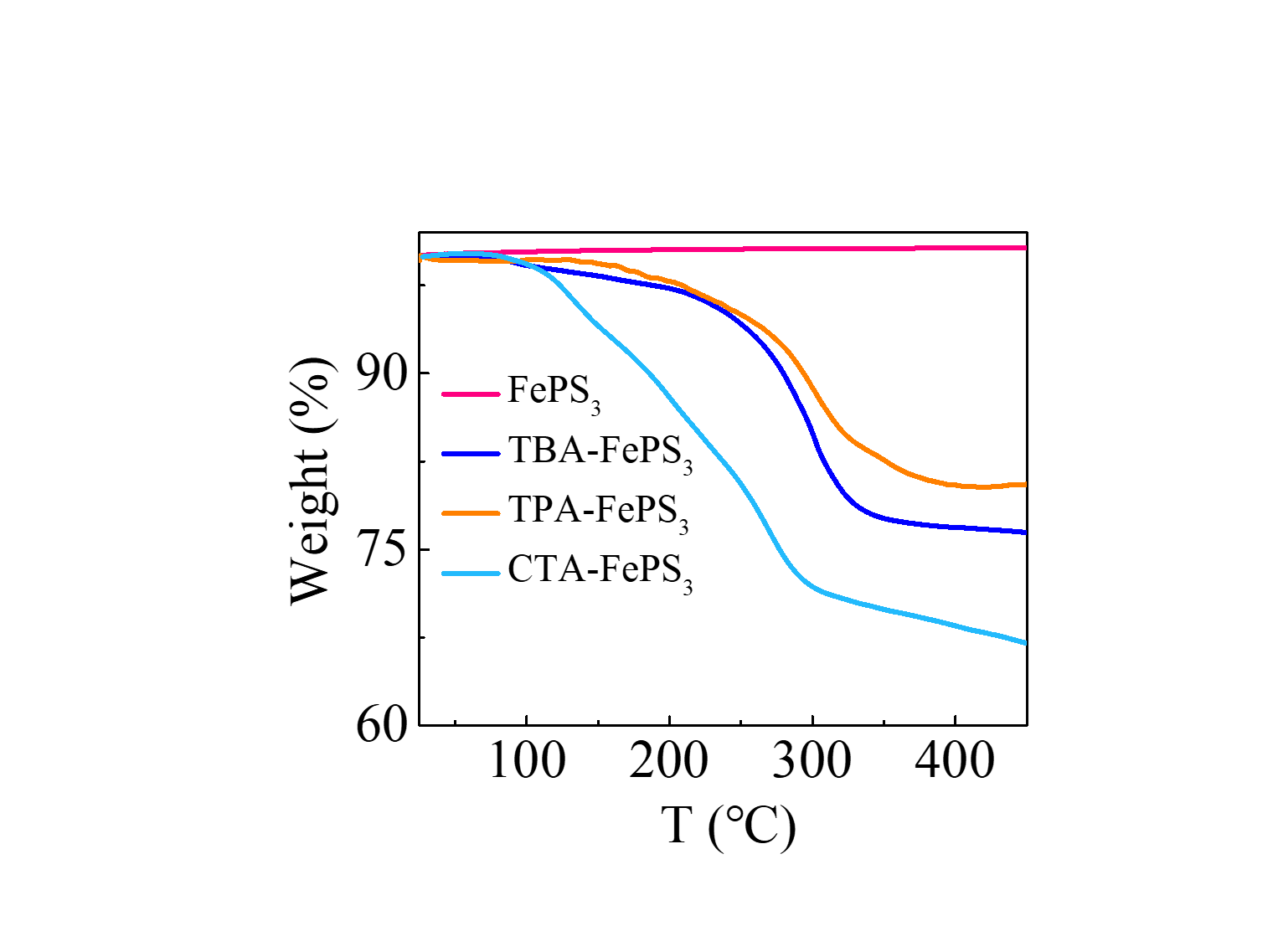


**Figure S4.** The TGA for intercalated TBA-FePS_3_, TPA-FePS_3_ and CTA-FePS_3_. The temperature range was 25 ℃ - 450 ℃ and the whole experiment was conducted under Nitrogen gas at a heating rate of 5 ℃ /min.


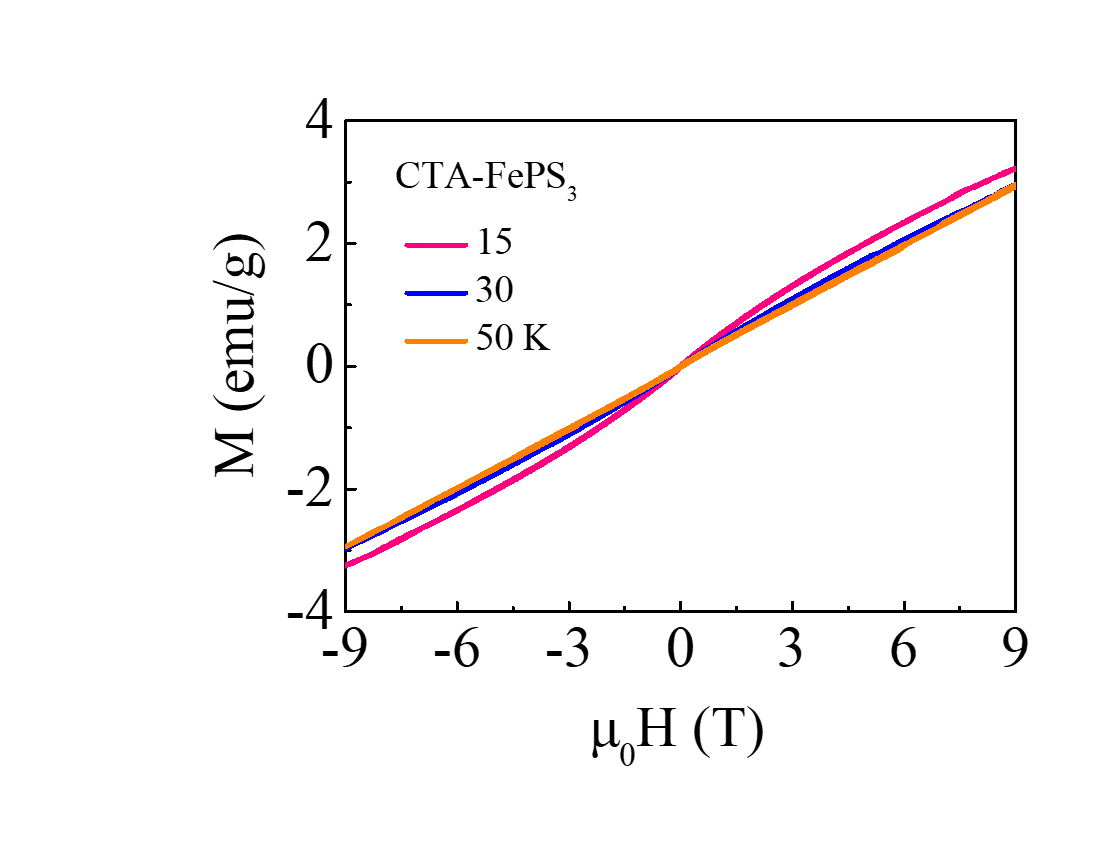


**Figure S5.** Magnetization versus magnetic field (*M*-*H*) of intercalated CTA-FePS_3_ under magnetic field *H* // *c** at different temperatures.


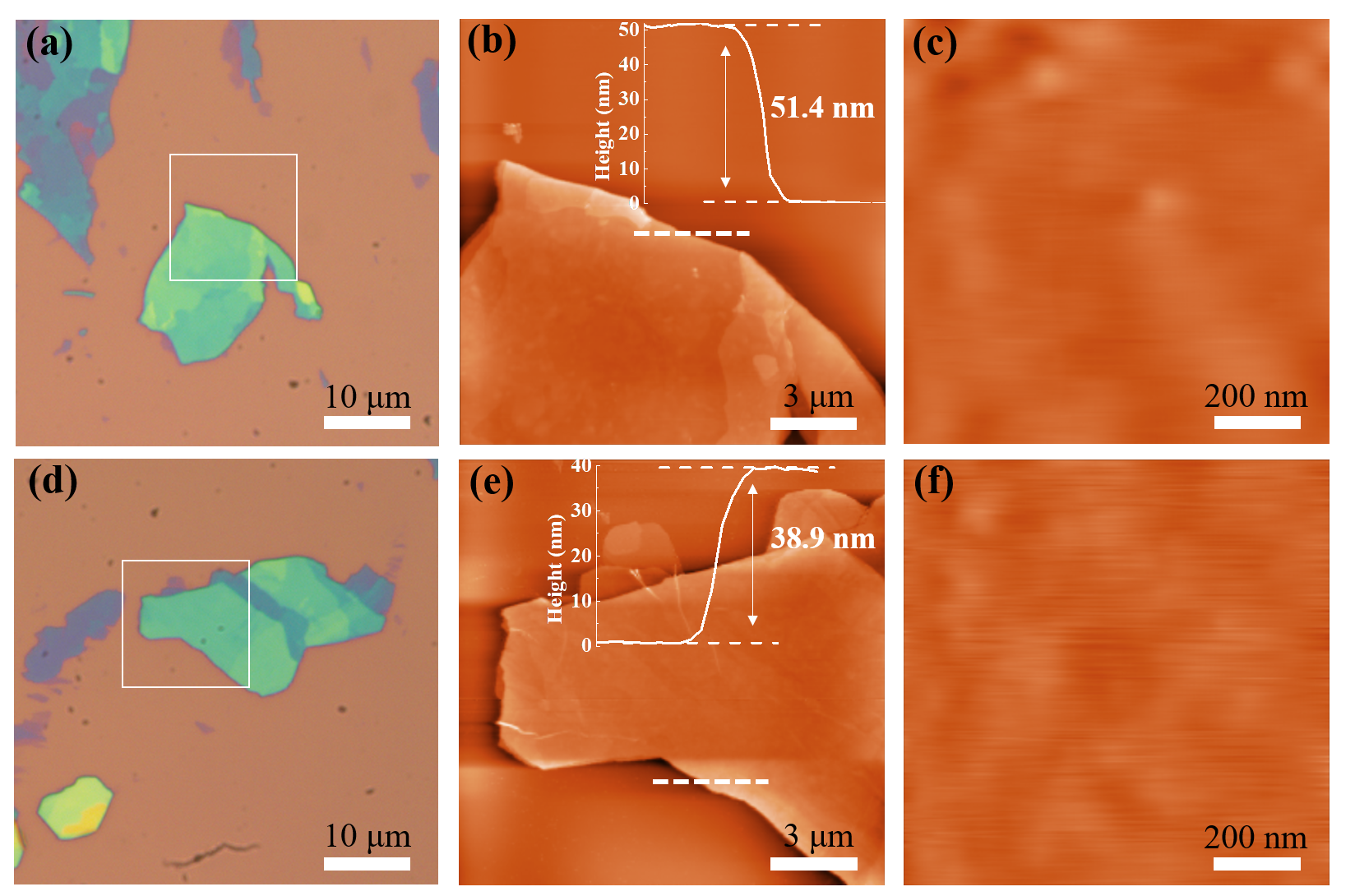


**Figure S6.** Morphologies of intercalated CTA-FePS_3_ and TBA-FePS_3_. (a-c) Optical image (a), AFM topography image (b) and enlarged AFM image (c) of CTA-FePS_3_, respectively. (d-f) Optical image (d), AFM topography image (e) and enlarged AFM image (f) of intercalated TBA-FePS_3_, respectively. Inserts in (b) and (e) are AFM height profiles of CTA-FePS_3_ and TBA-FePS_3_ corresponding to white dashed lines, respectively.


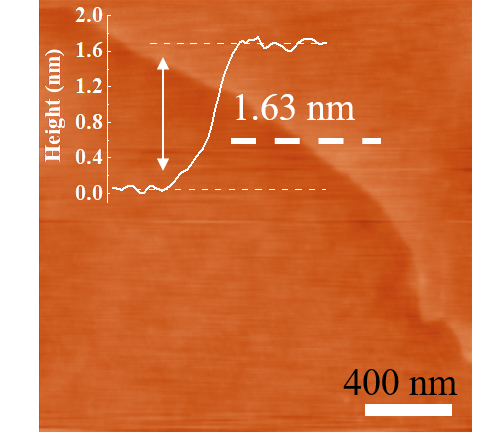


**Figure S7.** AFM image of exfoliated intercalated THA-FePS_3_ flake, showing a step height of ~1.63 nm. Inset shows a height profile along the white dashed line.


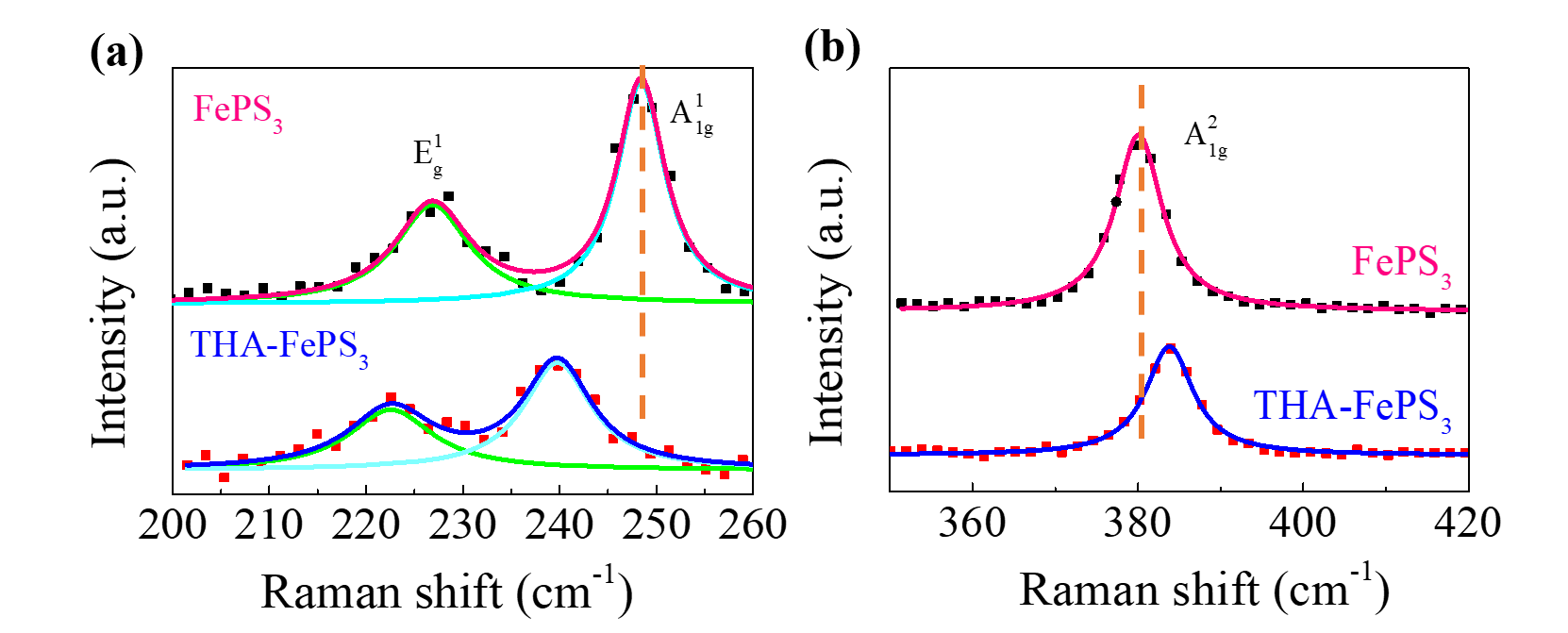


**Figure S8.** (a), (b) Detailed view of out-of-plane A_1g_ modes for bulk pristine FePS_3_ and intercalated THA-FePS_3_, where spectra are fitted with Lorentzian line shape.


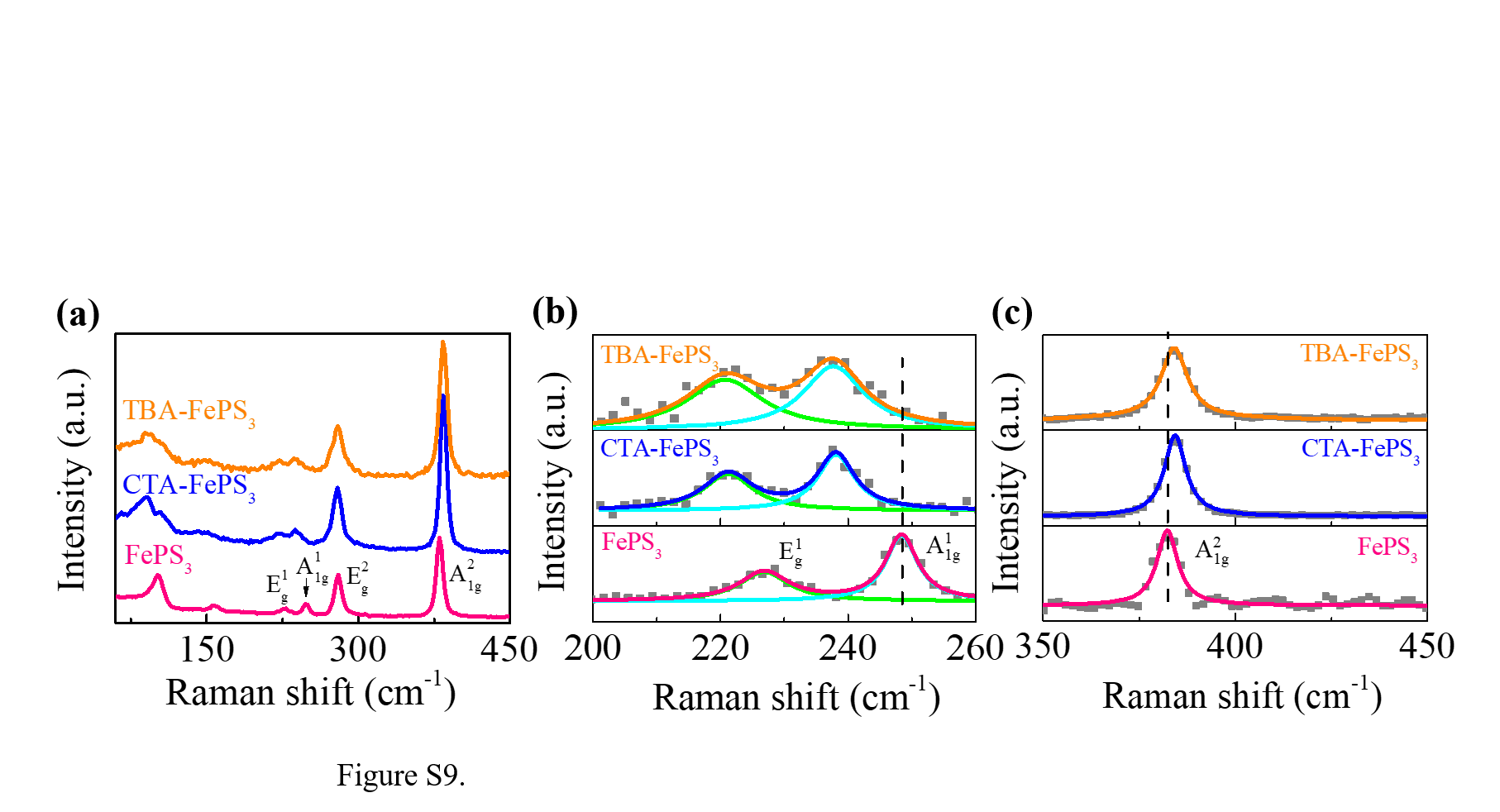


**Figure S9.** (a) Raman spectra of TBA-FePS_3_, CTA-FePS_3_, and FePS_3_. (b), (c) Detailed view of out-of-plane A_1g_ modes for pristine FePS_3_ and intercalated FePS_3_, where spectra are fitted with Lorentzian line shape.

To investigate the structural changes after intercalation, in-plane high-resolution transmission electron microscopy (HRTEM) and selected area electron diffraction (SAED) images of THA-FePS_3_ were performed. For the test, THA-FePS_3_ flakes were sonicated in ethanol, and then resulting suspension was dropped onto the TEM grid.


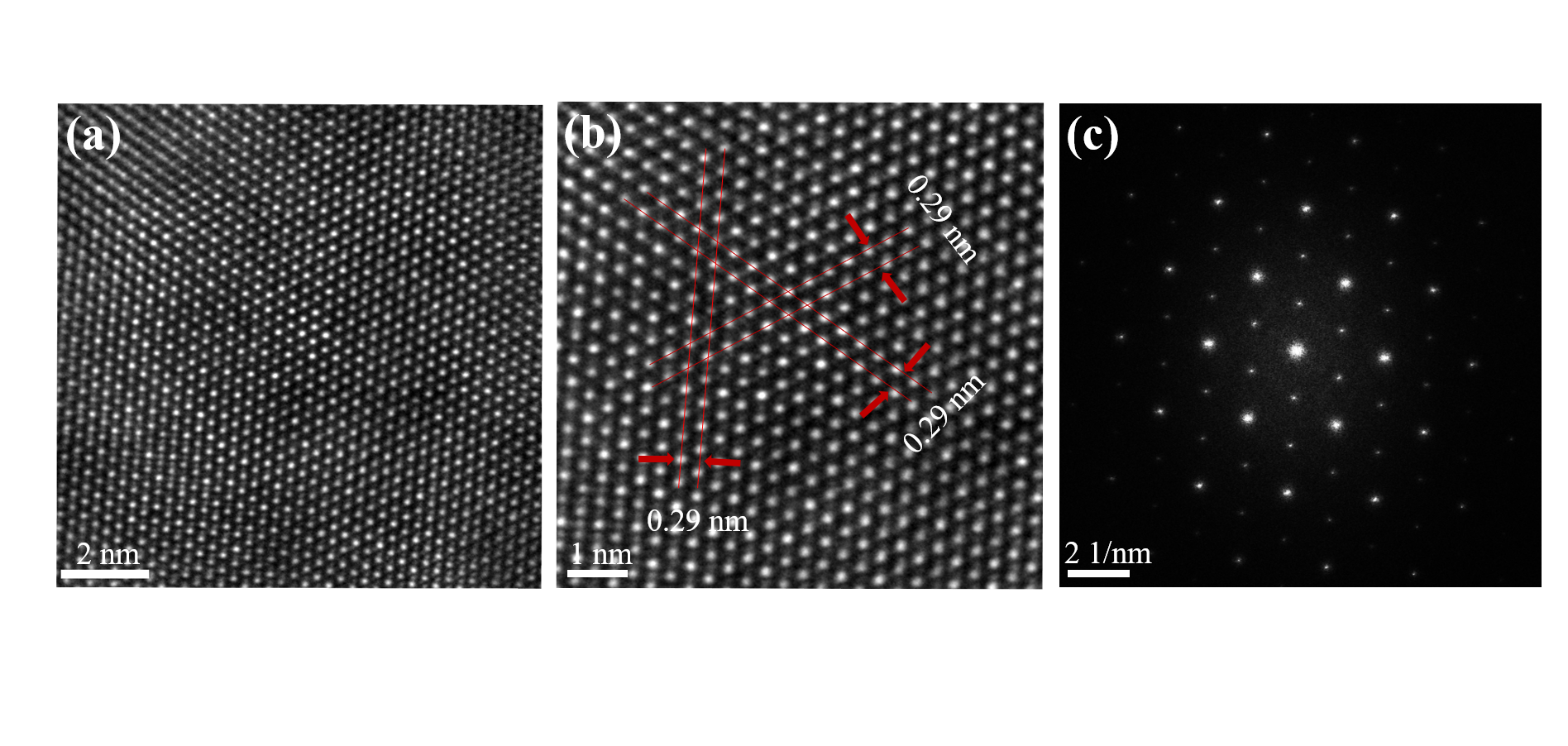


**Figure S10**. The in-plane HRTEM (a, b) and SAED images of THA-FePS_3_.

**Table S1.** Peak positions of binding energies in XPS spectra of Fe, S, P elements for pristine FePS_3_ and intercalated THA-FePS_3_

|  | Fe | | S | | P | |
| --- | --- | --- | --- | --- | --- | --- |
|  | 2*P*_3/2_ | 2*P*_1/2_ | 2*P*_3/2_ | 2*P*_1/2_ | 2*P*_3/2_ | 2*P*_1/2_ |
| FePS_3_ | 709.8 | 723.1 | 162.8 | 164.0 | 132.5 | 133.3 |
| THA-FePS_3_ | 708.9 | 722.2 | 162.1 | 163.3 | 131.7 | 132.5 |
| ΔE | 0.9 | 0.9 | 0.7 | 0.7 | 0.8 | 0.8 |

ΔE = E(FePS_3_)-E(THA-FePS_3_), the peak positions are fitted with CasaXPS software.


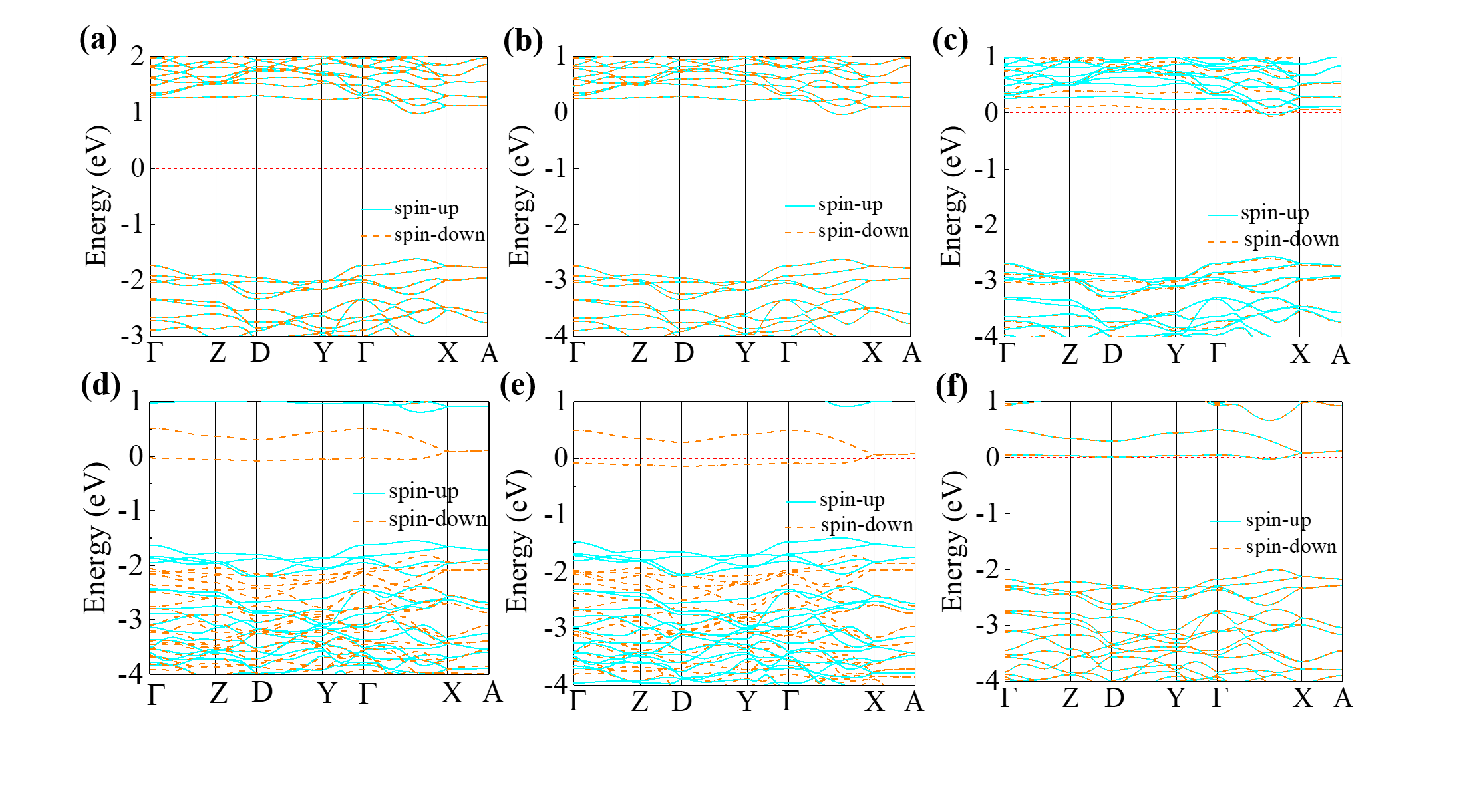


**Figure S11.** The band structures of FePS_3_ with doping concentrations of 0.0 (a), 0.2 (b), 0.3 (c), 0.8 (d), 0.9 (e), and 1.0 (f) electrons/cell.


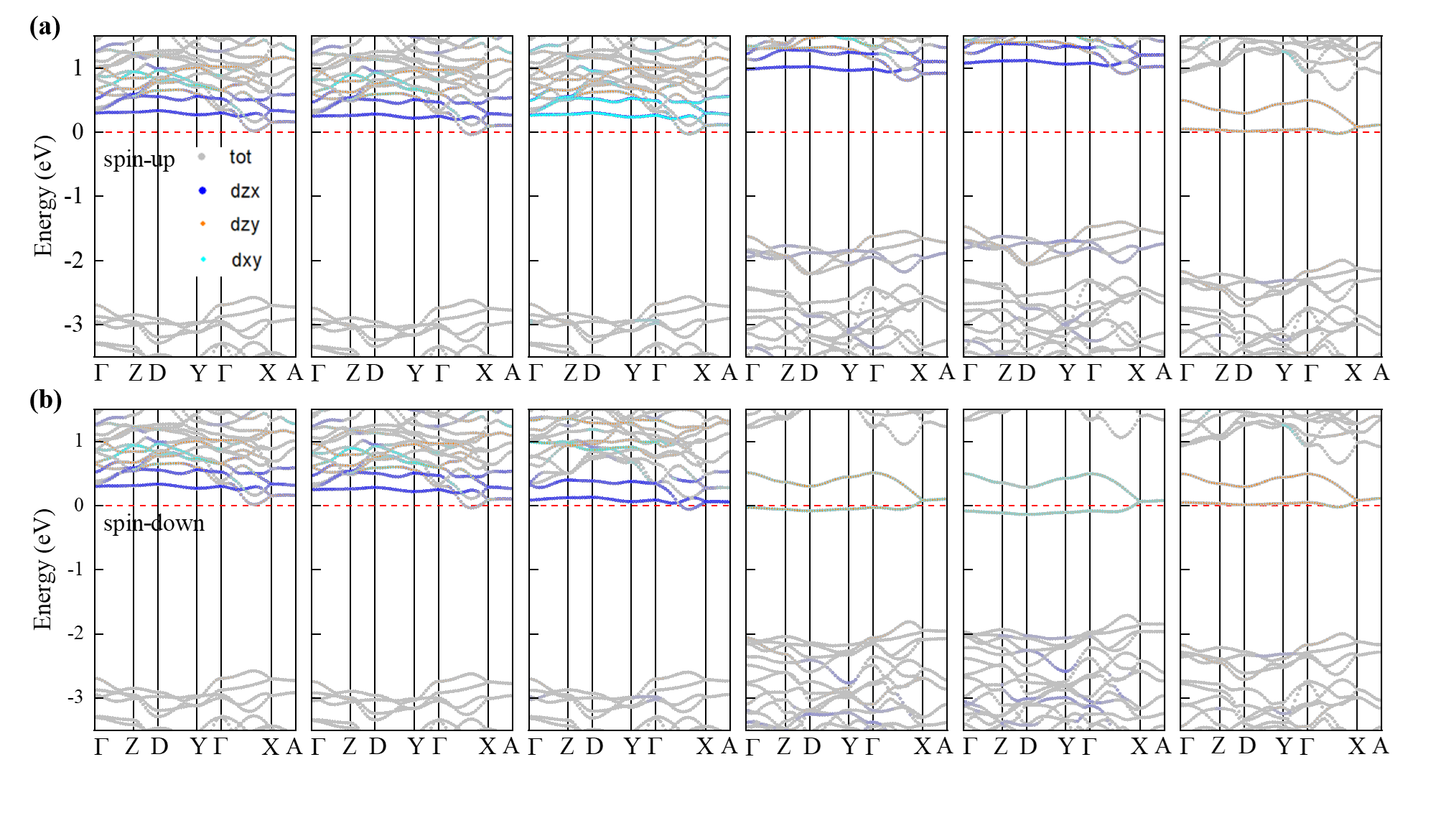


**Figure S12.** Orbital-resolved band structures of FePS_3_ for spin-up (a) and spin-down (b) configurations with doping concentrations of 0, 0.2, 0.3, 0.8, 0.9, and 1.0 electrons/cell from left to right. The colors of blue, orange and cyan represent the contributions of Fe *d*_zx_, *d*_zy_, and *d*_xy_ orbitals, respectively.

According to FePS_3_ cluster model,^[1]^ the calculated ground state is given as $\Psi_{g}=\alpha|d^{6}>+\beta|d^{7}\underline{L}>+\gamma|d^{8}\underline{L}^{2}>\lambda|d^{9}\underline{L}^{3}>\mu|d^{10}\underline{L}^{4}>$, where $\underline{L}$ indicates a ligand (sulfur) hole. The Fe^2+^ ion from the formal valence predicts an occupation number of 6, while we obtained 6.22 by calculating the total number of *d* electrons from the PDOS analysis (Figure S11). It is suggested that some contributions from $d^{7}\underline{L}$, $d^{8}\underline{L}^{2}$, $d^{9}\underline{L}^{3}$ and $d^{10}\underline{L}^{4}$ configurations are expected besides$d^{6}$. Furthermore, the magnetic moment of the Fe^2+^ is 3.6$\mu_{B}$, as opposed to the formal value of 4$\mu_{B}$. These results indicate that FePS_3_ can be regarded as a self-doping negative charge transfer insulator.


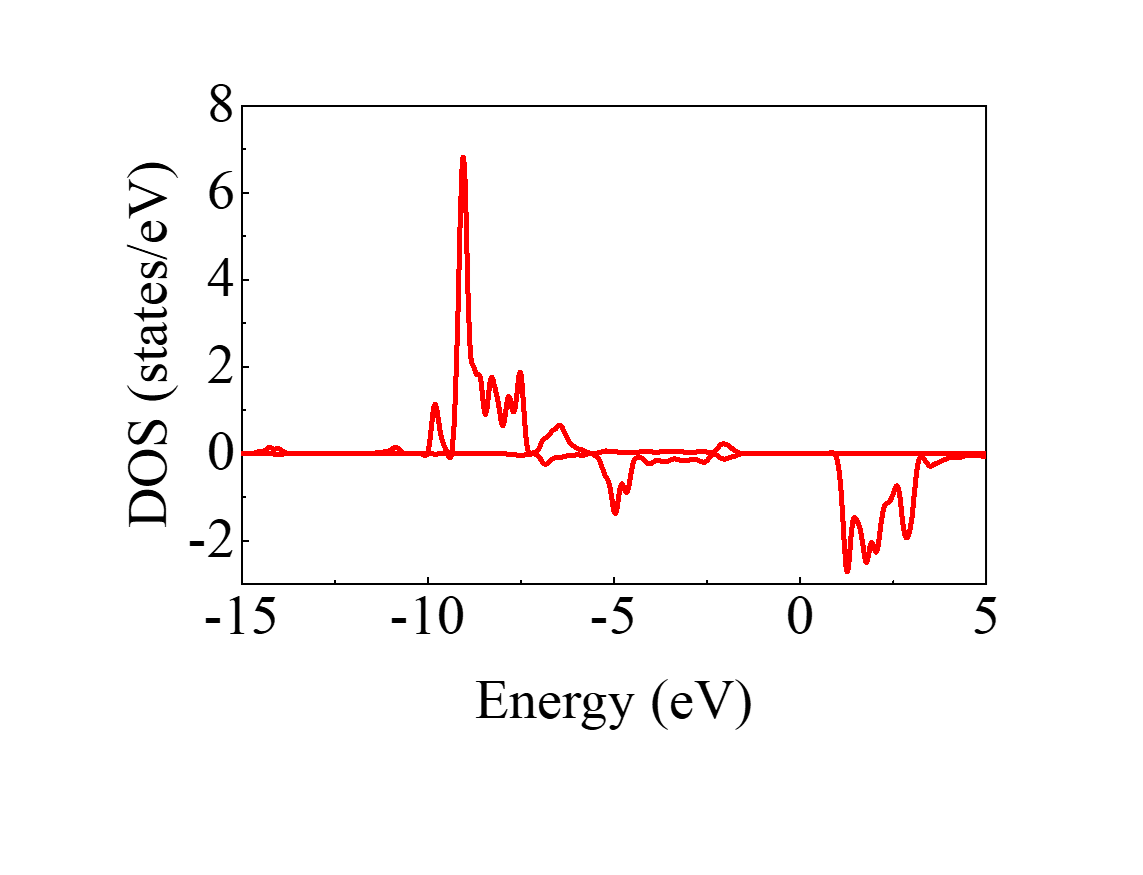


**Figure S13.** The density of states of Fe^2+^ ions in FePS_3_.


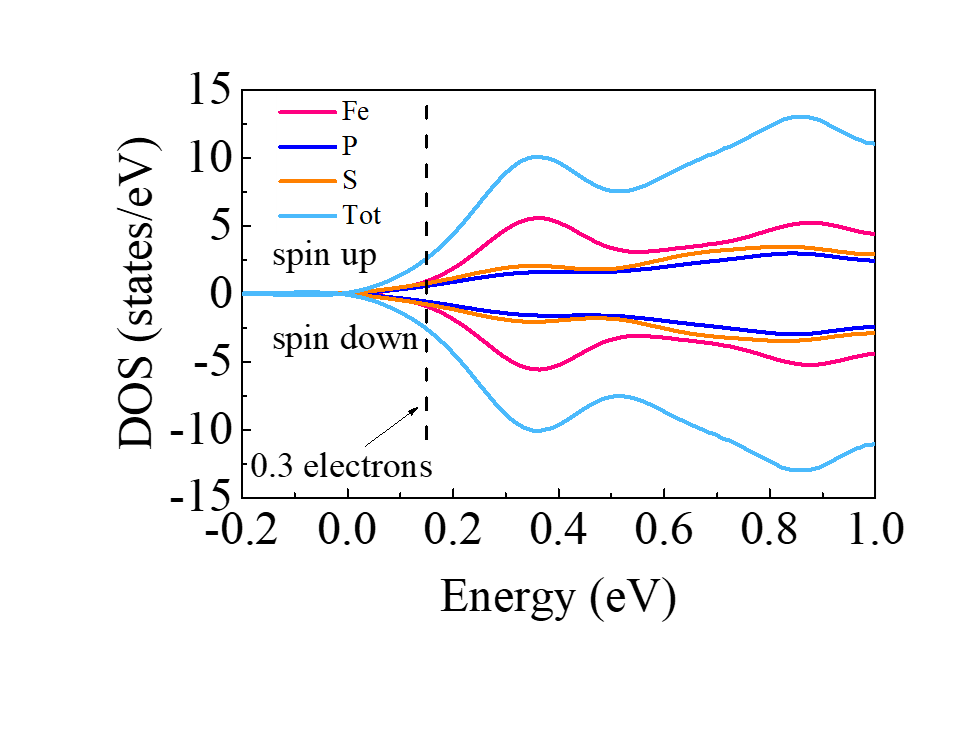


**Figure S14.** An enlarged conduction band of pristine FePS_3_. The vertical line represents the energy level of 0.3 electrons filled in the conduction band.

Table S2. The energies of four different magnetic states (nAFM, sAFM, zAFM, FM) with higher doping concentrations in FePS_3_. The energy of nAFM state is set to zero at each electron doping concentration.

| Doping level (e/cell) | E_nAFM_ (meV) | E_sAFM_  (meV) | E_zAFM_  (meV) | E_FM_  (meV) |
| --- | --- | --- | --- | --- |
| 1.0 | 0.00 | 45.39 | 0.89 | 2.05 |
| 1.1 | 0.00 | -36.43 | 56.89 | -36.42 |


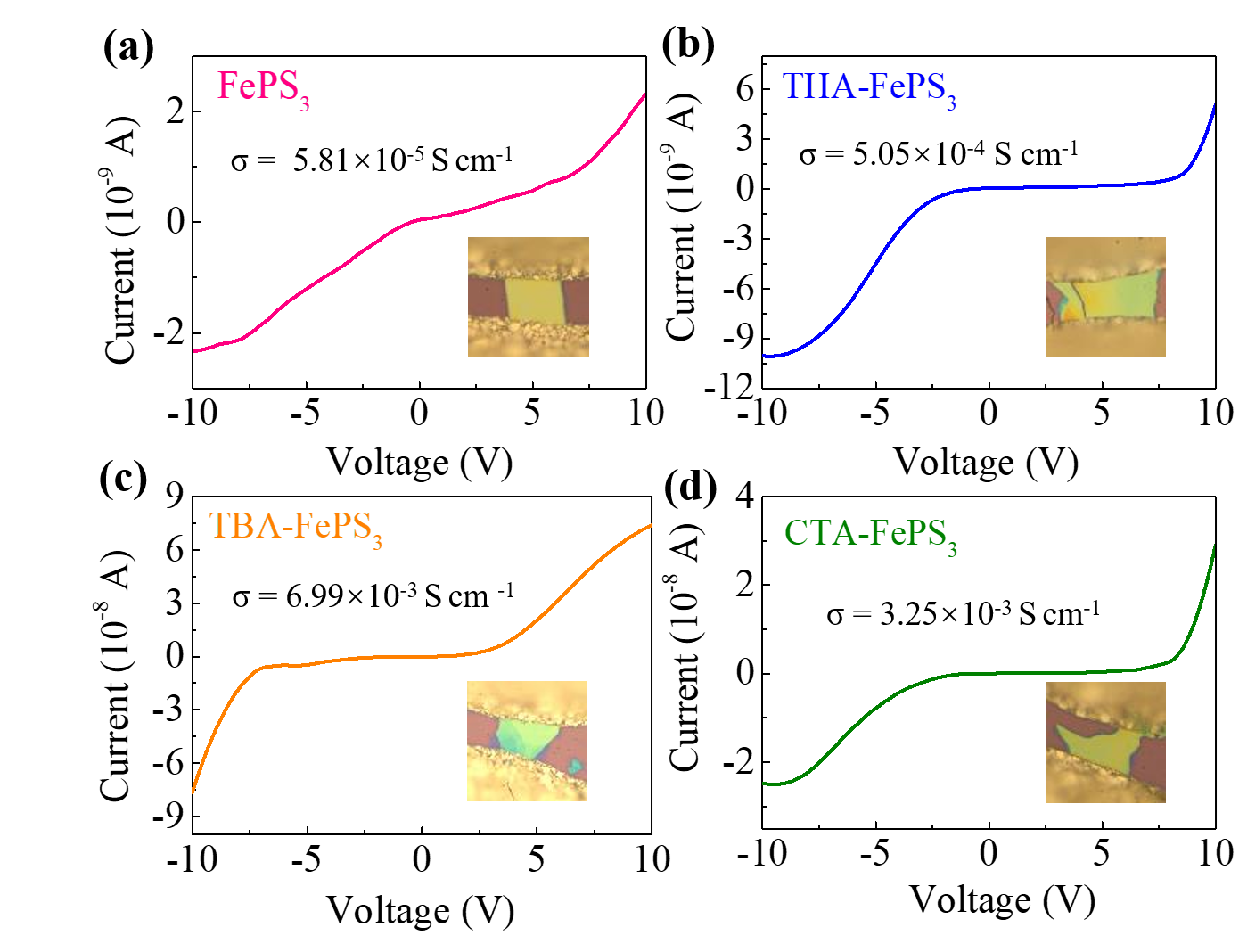


**Figure S15.** (a)-(d) Drain current versus drain voltage of pristine FePS_3_ (a), THA-FePS_3_ (b), TBA-FePS_3_ (c) and CTA-FePS_3_ (d). Inset in each figure shows the optical image of corresponding device.

It is challenging to quantitatively obtain the carrier density of intercalated FePS_3_ even after being doped due to the expanded volume composed of lots of loosely stacked flakes (Figure S16) and degraded properties caused from microfabrication processes. Therefore, we performed the electrical conductivity measurement on a single flake of the intercalated FePS_3_. Before making electrodes, we firstly performed the Raman spectra to confirm the successful intercalation of exfoliated flakes (Figures S8, S9).


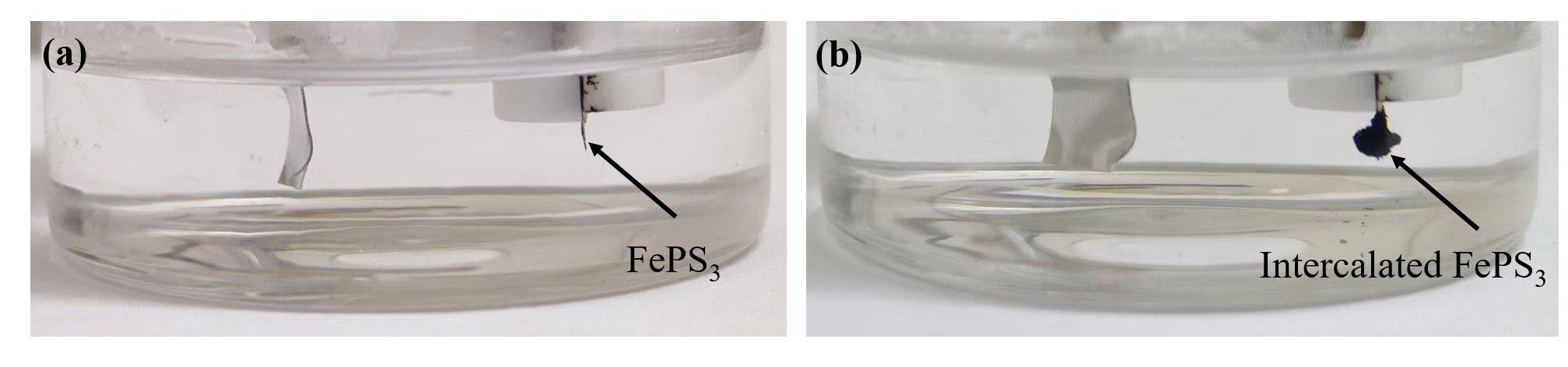


**Figure S16.** Photographs of FePS_3_ before (a) and after intercalation (b), showing a substantially large volume expansion.


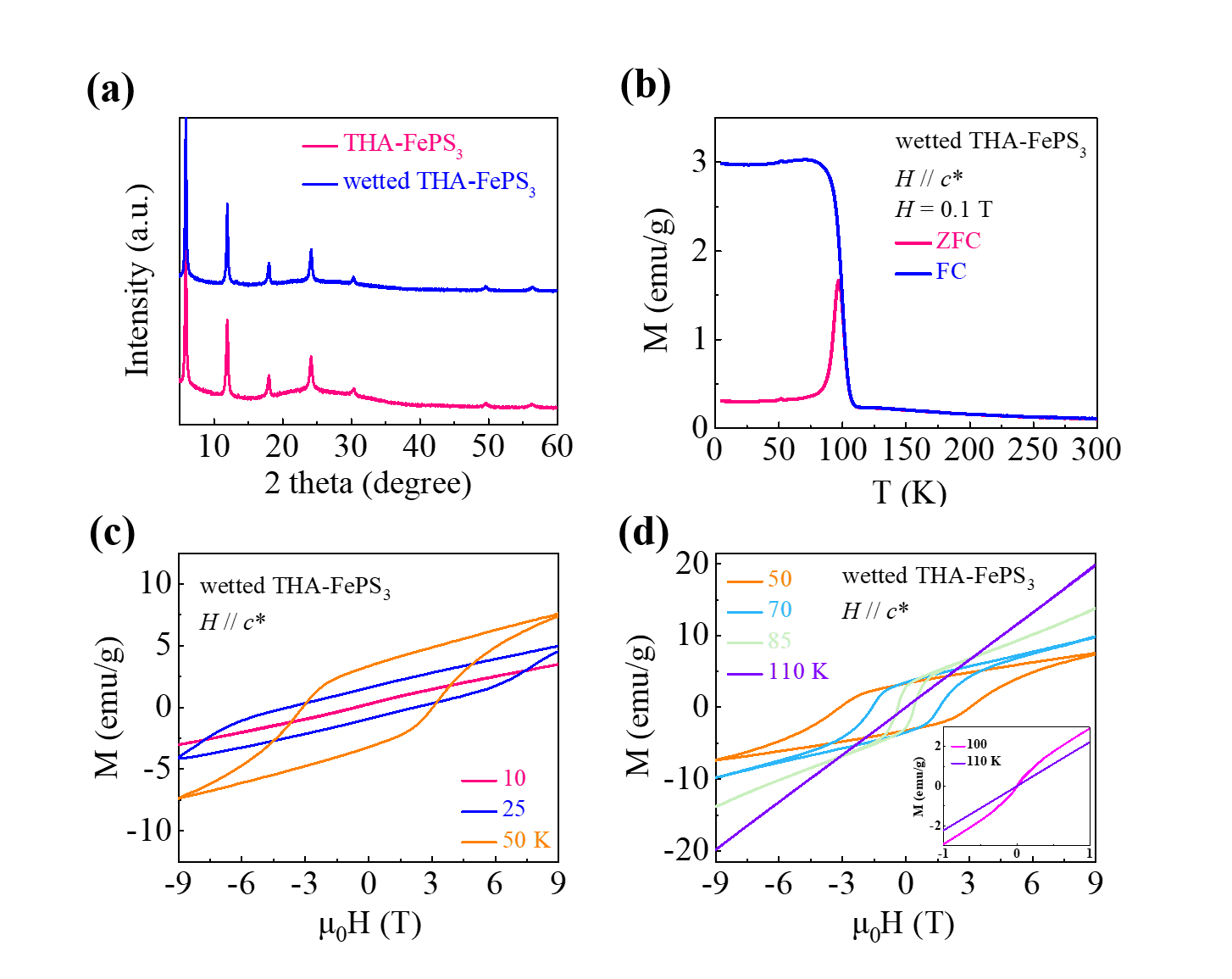


**Figure S17.** (a) The XRD patterns of THA-FePS_3_ and wetted THA-FePS_3_; (b) Magnetization versus temperature (*M*-*T*, b) of wetted THA-FePS_3_ under magnetic fields *H* // *c**. (c), (d) Magnetization versus magnetic field μ_0_H (*M*-*H*) at different temperatures under magnetic fields *H* // *c**. The inset of (d) shows the zoom-in image of the *M*-*H* curves at 100 K and 110 K.


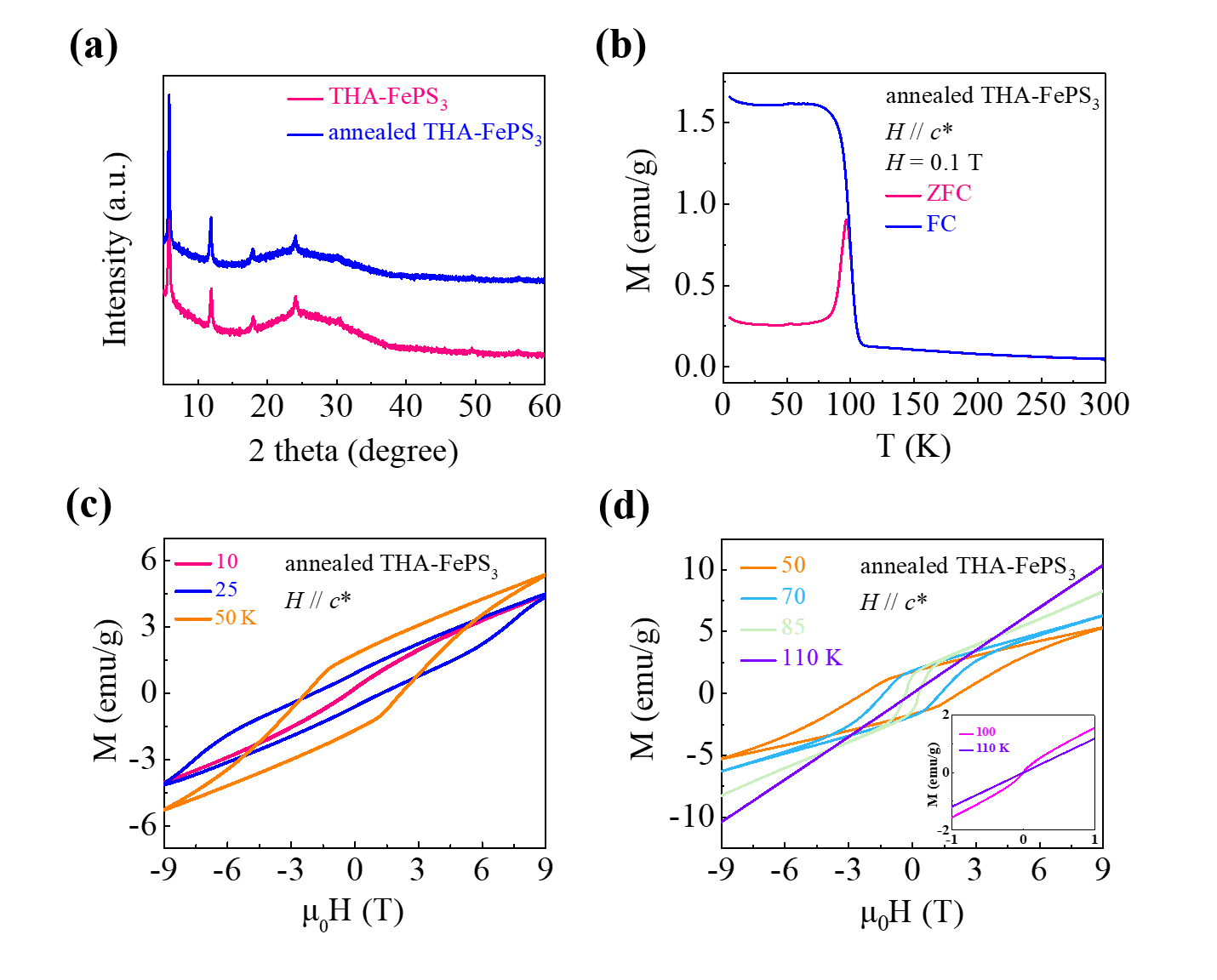


**Figure S18.** (a) The XRD patterns of the original THA-FePS_3_ and the annealed THA-FePS_3_; (b) Magnetization versus temperature (*M*-*T*, b) of annealed THA-FePS_3_ under magnetic fields *H* // *c**. (c), (d) Magnetization versus magnetic field μ_0_H (*M*-*H*) at different temperatures under magnetic fields *H* // *c**. The inset of (d) shows the zoom-in image of the *M*-*H* curves at 100 K and 110 K.

**Reference**

[1] S. Y. Kim, T. Y. Kim, L. J. Sandilands, S. Sinn, M.-C. Lee, J. Son, S. Lee, K.-Y. Choi, W. Kim, B.-G. Park, C. Jeon, H.-D. Kim, C.-H. Park, J.-G. Park, S. J. Moon, T. W. Noh, *Phys. Rev. Lett.* **2018**, 120, 136402.
